# The Pleidae (Hemiptera, Heteroptera) of Thailand, with the descriptions of two new species and a discussion of species from Southeast Asia

**DOI:** 10.3897/zookeys.973.54026

**Published:** 2020-10-05

**Authors:** Jerry L. Cook, Robert W. Sites, Akekawat Vitheepradit

**Affiliations:** 1 Department of Biological Sciences, Sam Houston State University, Huntsville, TX 77341, USA Sam Houston State University Huntsville United States of America; 2 Enns Entomology Museum, University of Missouri, Columbia, MO 65211, USA University of Missouri Columbia United States of America; 3 Department of Entomology, Kasetsart University, Bangkok 10900 Thailand Kasetsart University Bangkok Thailand

**Keywords:** distribution, *
Paraplea
*, taxonomy

## Abstract

The family Pleidae is represented in Thailand by four species in the genus *Paraplea*. Two of these species, *P. frontalis* and *P. liturata*, are widespread and relatively common in Southeast Asia. Two other species, *P. lateromaculata* Cook, **sp. nov.** and *P. melanodera* Cook, **sp. nov.**, are described and only known from Thailand. Full descriptions are provided for all four species. The distributions of these species are discussed, with an emphasis on Thailand. *Paraplea frontalis*, *P. liturata*, and *P. lateromaculata* Cook, **sp. nov.** are relatively widespread within Thailand and have overlapping distributions whereas *P. melanodera* Cook, **sp. nov.** appears restricted to small brackish ponds near western coastal areas of peninsular Thailand.

## Introduction

The family Pleidae in Southeast Asia is represented only by species in the genus *Paraplea*. *Paraplea areolata* Paiva, 1918 was described from Myanmar (Burma,) and *P. davaoensis* Miyamota, 1981 and *Paraplea sobrina* (Stål, 1860) are known from the Philippines. Two species, *P. frontalis* (Fieber, 1844) and *P. liturata* (Fieber, 1844) are likely widespread in Southeast Asia, and also occur outside this region. In Southeast Asia, *P. frontalis* and *P. liturata* have been recorded from Indonesia, Myanmar (Burma), West Malaysia, Singapore, Taiwan, and Thailand. *Paraplea frontalis* is also known from China, India, Sri Lanka, and Taiwan, and *P. liturata* from Australia, Japan, and New Caledonia. *Paraplea vittifrons* (Horváth, 1919) is known from the type specimen from the Aru Islands of Indonesia, which is in maritime Southeast Asia.

Members of the family Pleidae are rarely collected. These are very small aquatic bugs that are often overlooked in nature and collections, and they occur in habitats that are rarely sampled. The most common habitat where pleids occur is in slow moving or stagnant waters, with rich vegetation (Andersen and Weir 2004), although this may not be true for some regions, such as Thailand. Besides being overlooked and occurring in a habitat that is rarely collected, there has been little recent research emphasis on this family. Chen et al. (2006) recorded aquatic Hemiptera in Thailand, and identified *P. frontalis* and *P. liturata* in northeastern Thailand. Herein we report results of extensive collections throughout Thailand and describe two new species of *Paraplea*, as well as report the distribution of the species of *Paraplea* of Thailand.

The taxonomy of species in Pleidae is based primarily on a few key characters of uncertain taxonomic importance. Traditionally, genera could be identified solely on the index of their tarsal segments (Cook 2011). All known pleid species have three tarsal segments on their metathoracic leg. Species with two tarsal segments on their prothoracic and metathoracic legs are included in *Paraplea*, whereas those with two prothoracic tarsal segments and three mesothoracic tarsal segments are placed in *Neoplea*. Species with three tarsal segments on all legs are in *Plea* or *Heteroplea*, the latter having a callus posteriorly on the head. However, this classification may be artificial as there has been no phylogeny of the family. Other characters that appear to be valid for taxonomic purposes include the tooth pattern of the ovipositor (Sublett and Cook 2015), profile of the sternum, widths of the pronotum and scutellum, state of body sculpturing, mele and female opercula, mele parameres, and form of the clavus (Drake and Chapman 1953). Cook (2017) added indices of body shape, an ocular index, pronotal index, scutellar index and scutellar length index to the evaluation of species in Pleidae. The descriptions herein use all of these characters to delineate species.

The genus *Paraplea* is the most widespread genus in Pleidae with 19 valid species. Two species are from the New World, known primarily from the Caribbean and the southeastern United States; four species are from Africa; two from India; two from Australia; three from Japan or Taiwan; and six from Southeast Asia. With the addition of the two species from Thailand described in this study, this region is clearly the most diverse for *Paraplea*. However, there are likely many more species that remain unknown and await discovery and description.

## Materials and methods

Most specimens for this study were field collected by co-authors Sites and Vitheepradit and their colleagues. Photographs of these collection sites, identified as L-numbers, are available in a Locality Image Database via a link from the internet site of the Enns Entomology Museum, University of Missouri. Other specimens were from previously collected materials deposited in the Snow Entomology Collection, University of Kansas (**SEMC**) and the United States National Museum (**USNM**). The recently collected specimens for this study are housed primarily in the Enns Entomology Museum, University of Missouri (**UMC**) and Sam Houston State University Natural History Collections (**SHSU**). Maps using data reported in the text were produced with SimpleMapper (Shorthouse 2010). Observations and measurements were made using an Olympus SZX16 microscope with an ocular micrometer and a Keyence VHX-6000 digital microscope. In total, 1279 adult specimens of Thai Pleidae were examined for this study, with 948 used for measurements due to their overall condition and orientation. Indices used for the description are:

**BSI** Body Shape Index (body width/body length) x 100

**OI** Ocular Index (narrowest width between eyes/width of head across the eyes) x 100; width between eyes is taken anteriorly in dorsal view, width of head is at widest point including eyes

**PI** Pronotal Index (length of pronotum/width of pronotum) x 100

**SI** Scutellar Index (scutellum width/scutellum length) x 100

Description of the ovipositor (gonapophyses) follows the terminology of Sublett and Cook (2015).

## Results and discussion

Four species were found in Thailand, two previously described and two new. Although the two previously described species, *P. frontalis* and *P. liturata*, are relatively well known, they lack a complete modern description. Both were partially redescribed by Lundblad (1933) but not all meaningful taxonomic characters were addressed. All species of *Paraplea* found in Thailand are described below. Two species are new descriptions and two species are supplementary redescriptions.

### Paraplea frontalis

Taxon classificationAnimaliaHemipteraPleidae

(Fieber, 1844)

696A8DF2-43DB-5E88-9638-D425C58C67DB

**Figure 1.**
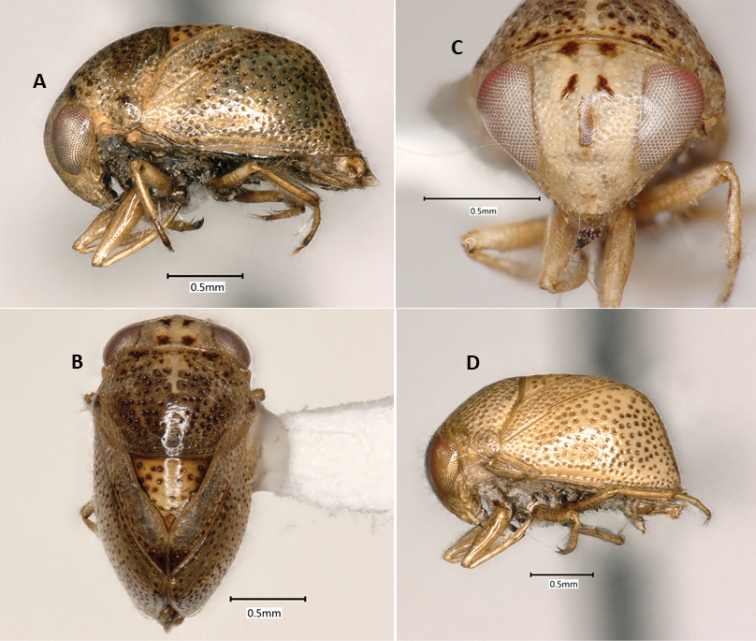
*Paraplea frontalis* specimens from Thailand. Male with most typical coloration in (**A**) lateral view, (**B**) dorsal view, and (**C**) frontal view. (**D**) Female showing an alternatively colored form in lateral view.

**Figure 2.**
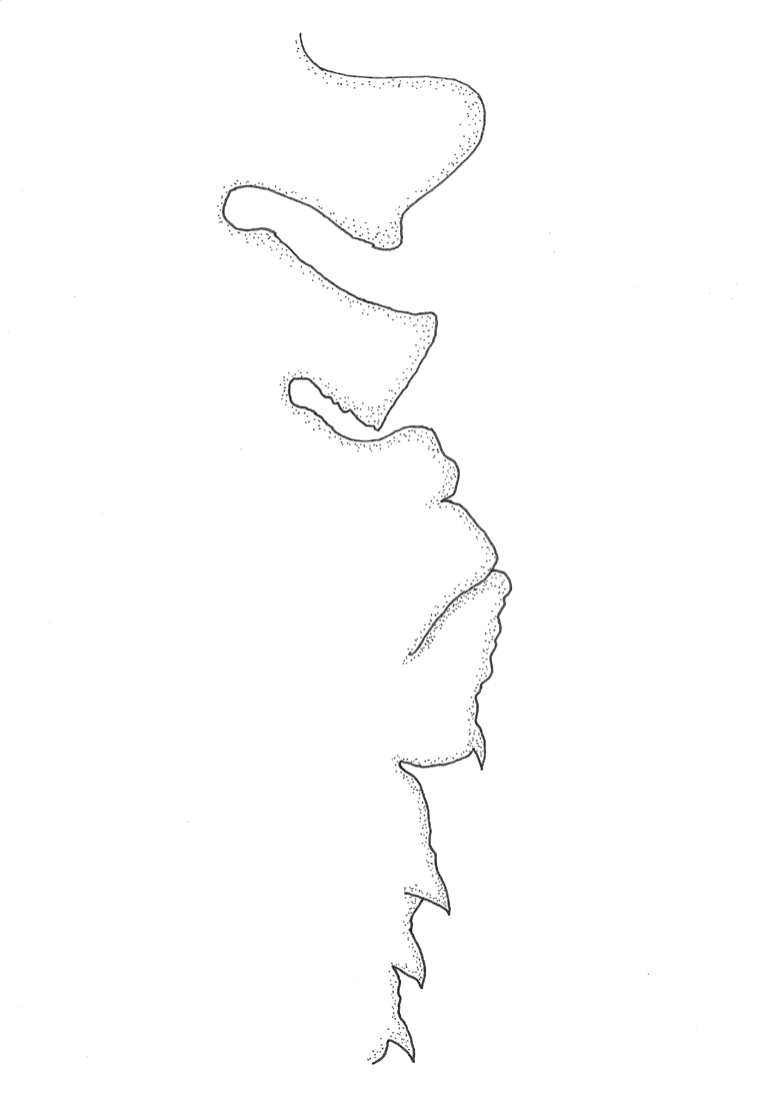
Ventral keel of *Paraplea frontalis*, anterior region to the left, ventral to the top.

**Figure 3.**
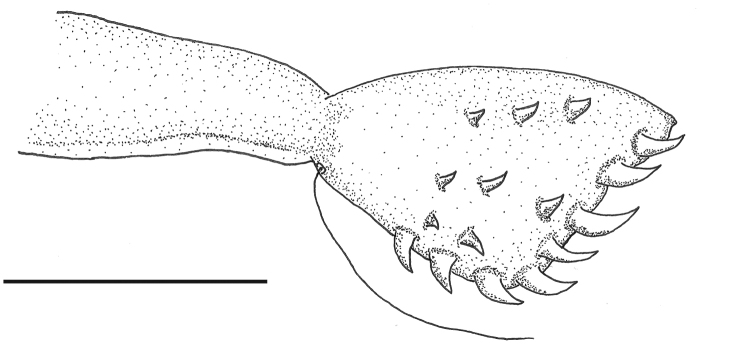
Ovipositor of *Paraplea frontalis* with gonapophysis 1 to right and gonapophysis 2 to left. Scale bar: 0.1 mm.

**Figures 4–5.**
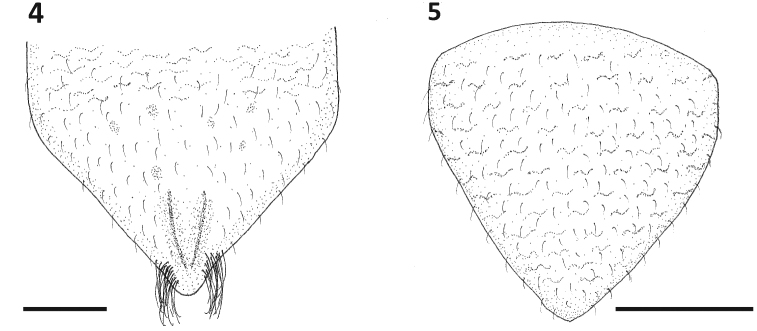
Genital plates of *Paraplea frontalis*. **4** female, **5** mele. Scale bar: 0.2 mm.

Figures 1 , 2 , 3 , 4–5 , 24A

= Ploa frontalis Fieber, 1844: Entomol. Monogr. p. 18.
= Plea frontalis: (Kirkaldy, 1898): Wien. Entomol. Zeit. 17: 141.
= Plea pelopea Distant, 1911: Fauna Brit. India 5: 336–337 (synonymized by Lundblad 1933: 138).
= Plea quinquemaculata Lundblad, 1933: Arch Hydrobiol. 12: 135–138 (synonymized by Nieser 2004: 82).
Plea (Paraplea) frontalis : Esaki and China 1928: Rev. Esp. Entomol. 4: 166 (subgenus description).
Paraplea frontalis : Drake and Maldonado-Capriles 1956 (elevation to genus) 51: 53.

#### Remarks.

*Paraplea frontalis* was described as *Ploa frontalis* by Fieber (1844) for specimens collected in the East Indies. The original description was not extensive and relied heavily on coloration, which has proven to be a somewhat variable character in pleids. However, this description included a documentation of the distinctive markings of the face and vertex that is found in most specimens of this species. The figures provided with the original description are not very helpful in distinguishing *P. frontalis* from other pleid species. No types were designated by Fieber; however, the distinctive markings of the head made associations with subsequent collections possible with a relatively high degree of certainty. Kirkaldy (1898) reported on a specimen from Rangoon (now Yangon) in present day Myanmar (Burma), and in this and his later publication (Kirkaldy 1904), he followed Leach (1817) in putting all of Pleinae into the genus *Plea*, thus changing the name of this species to *Plea frontalis*. Kirkaldy (1904) also included a range extension of the species into Bengal, which is now in Bangladesh, although it is possible that he did not distinguish between West Bengal and East Bengal, leaving uncertainty to the exact region, which would now be in either India or Bangladesh, respectively. Kirkaldy also reported specimens from Pondicherry (India) and Cochin, China, which is now in Vietnam. Kirkaldy did not provide a description of the specimens he included in *P. frontalis*. Distant (1906) provided an English translation of the original description by Fieber but stated that he was unable to view any specimens of the species. Distant (1910) described a species from Calcutta and Madupur, West Bengal, India as *Plea pelopea* based primarily on coloration, including having a head with four dark spots and commented that he thought this could be an “extreme variety” of *Plea pallescens*. Plea (Paraplea) pelopea was considered by Lundblad (1933) to be the same as *P. frontalis*, although there was no type available for comparison. Lundblad (1933) determined that all previous treatments of *P. frontalis* did not allow for precise identifications. He commented on the most common head marking of having five dark markings and provided drawings of the head, antenna, legs, pronotum, sternal crest and parameres, but he did not provide a complete re-description. Lundblad (1933) also described a new species, Plea (Paraplea) quinquemaculata, which is now considered a synonym of *P. frontalis* (Nieser 2004). Benzie (1989) redescribed the species using specimens from Sri Lanka, including many of the characters used for modern descriptions in this family. Although all of these forms are now included as *P. frontalis*, it is possible that this represents a species complex. Below is a supplemental description of the species, based primarily on specimens from Thailand that fit within *P. frontalis* as it is currently defined.

#### Type information.

No holotype is designated. Fieber (1844) reported that the description was made from specimens collected by Dr. Helfer in the East Indies, although the exact location in this region of Southeast Asia is not documented in the literature. A specimen of Pleidae collected by Dr. Helfer is deposited the National Museum in Prague (NMPC) that could give an indication about the area where the original *P. frontalis* was collected (Nico Nieser communication). This specimen could become a neotype if it is determined to be *P. frontalis*, however this specimen has not yet been evaluated.

#### Supplementary description.

All measurements are given in millimeters from 456 adult specimens from throughout Thailand as reported in the distribution portion of this paper. Specimens used for this description are deposited at SEMC, SHSU, and UMC. Other specimens from Thailand and areas outside Thailand were surveyed but were not included in measurements due to condition or availability.

***Body size*.** Total length, 1.89–2.32 (average 2.07) in Thailand specimens. Fieber (1844) reported total length of 2, but Lundblad (1933) reported that the species could reach 2.3 to 2.5. Greatest body width in Thailand specimens, 0.92–1.28 (average 1.04); BSI, 50–58.

***Color*.** Color may vary among individuals (Fig. 1A, D). Base color of body ranges from light brown to almost cream-colored, some with light honeycomb pattern, especially on pronotum; punctures throughout body usually with dark center; scutellum usually golden-tan; legs light brown; sternum and venter darker brown; eyes red to golden to silver in dried specimens, dark blotches seen in various places on some specimens, distinctive dark spots on face and vertex (Fig. 1C).

***Head*.** Head (Fig. 1C) generally light brown to cream colored with dark brown markings, mouthparts dark brown. Face and vertex normally with distinctive markings consisting of pair of spots near vertex, pair of spots between top margin of eyes and vertical line along midline of face, between middle of eyes; markings can differ between individuals; 64% with dark bar and four spots, 25% with light brown bar and four spots, 10% with light brown bar and two spots, 1% with either dark or light brown bar only (see Benzie 1989, Fig. 12 for diagram showing differences). Antenna three-segmented, usually hidden from view below eye. Head size similar among Thailand specimens, head width at widest point including eyes 0.92–1.04, head width at narrowest point between eyes, 0.47–0.56, OI 49–55.

***Pronotum*.** Base color ranging from cream to light brown, honeycombing apparent in some specimens; most have visible central lighter colored vertical band without punctures (Fig. 1B); slight humeral bulge present laterally; posterior margin with thick sclerotized border; punctures relatively large, 0.03–0.05, with dark centers; pronotum 1.01–1.17; length 0.62–0.79; PI 61–75 (Fig. 1B).

***Wings*.** Complete to posterior; punctures generally in irregular rows (0.02 in diameter) (Fig. 1A); underlying honeycomb structure sometimes present; claval suture distinct, complete; scutellum with distinct, dark punctures, more widely spaced than other punctures (Fig. 1B); scutellum base color often golden compared to tan base of hemelytra (Fig. 1B), although sometimes both cream colored (Fig. 1D); yellowish–brown spot at terminal angles of corium often present as reported in original description, but not always readily apparent, posterior half rarely brownish as in original description; lateral view shows distinct dorsal horizontal shape and posterior near vertical aspects with a transition of nearly 90° (Fig. 1A, C); scutellum slightly wider than long (Fig. 1B), scutellum length 0.46–0.57; scutellum width 0.54–0.66; SI 104–120. Hind wings membranous, fully developed, completely concealed by hemelytra.

***Legs*.** Typical leg measurements: prothoracic leg coxa 0.07, trochanter 0.11, femur 0.45, tibia 0.34, first tarsomere 0.02, second tarsomere 0.16,pretarsal claw 0.10; mesothoracic leg coxa 0.04, trochanter 0.18, femur 0.39, tibia 0.25, first tarsomere 0.02, second tarsomere 0.16, pretarsal claw 0.08; metathoracic leg coxa 0.04, trochanter 0.17, femur 0.47, tibia 0.53, first tarsomere 0.04, second tarsomere 0.18, third tarsomere 0.19, pretarsal claw 0.13 (see Benzie 1989, Fig. 9 for leg shapes).

***Median ventral keel*.** Thoracic portions distinct from each other; prosternal keel rounded with posterior blunt tooth; mesothoracic keel almost rectangular; metathoracic keel irregularly shaped, somewhat in lobes, including posterior projecting small tooth, closely associated with abdominal keel, appearing almost fused; thoracic segments relatively similar between specimens. Abdominal keel variable, usually partially fused between segments, usually with four distinct teeth corresponding with first four abdominal segments. Figure of typical Thailand specimen in Fig. 2, but also see drawing by Lundblad (1933, fig. 44H).

***Characters of female*.** Ovipositor roughly triangular in shape, 0.25 in length, with wide side apically (gonapophysis 1) at end of rectangular shaft (gonapophysis 2) (Fig. 3); six distinct teeth along posterior border (apical row) plus two teeth on ventral border (ventral 1 and 2), three rows of small teeth away from apex, two primary, three secondary, and usually three tertiary, although there is variation in number of tertiary teeth; one long hair on ventral side where triangular apex of gonapophysis 1 and basal rectangle of gonapophysis 2 meet. Subgenital plate as in Fig. 4; wider than long; width ~ 0.67, length ~ 0.41; faintly rugose in basal one third; tufts of relatively long hairs on each side near apex; short inconspicuous hairs throughout genital plate darker v-shaped prominence in center, near apex.

***Characters of male*.** Aedeagus bulbous and somewhat asymmetrical in the typical fashion of the family; operculum (subgenital plate) generally triangular, slightly wider than long (Fig. 5), width ~ 0.46, length ~ 0.41; lightly rugose throughout; with short hairs throughout.

#### Distribution.

*Paraplea frontalis* is a relatively widespread species in Thailand (Fig. 24A) as well as other parts of Southeast Asia. This study adds the first records from Laos.

#### Material examined.

Hong Kong: New Territories, Yuen Long, 23 II 1971, P. & P. Spangler (5 specimens USNM). India: Pondicherry, Maindeon, 1901 (4 specimens USNM); India, Tanquebor (South India) 1951, P. S. Nathan (1 specimen USNM); Karikal, VII 1956, P. S. Nathan (5 specimens USNM). Laos: Vientaine, along Mekong River, 17°58'02.6"N, 102°36'17.6"E, 6 VIII 1997 Wolfgang G. Ulrich (1 specimens USNM). Malaysia: Penang, University of Sains Malay, 29 I 1983, H. C. Chapman (15 specimens USNM). Myanmar: Rangoon Burma (= Yangon, Myanmar) Kemmencline 10 I 1927 (2 specimens USNM); Rangoon Burma (= Yangon, Myanmar) Kemmencline 29 I 1927 (4 specimens USNM). Singapore: 1 IX 1955. Marshall Laird (8 specimens SEMC). Thailand: **Ayutthaya Province**: roadside pond ca. 5 km E of Ayutthaya, 2 VII 1997, L-126, R. W. Sites (2 specimens UMC). **Bangkok Province**: 9–10 V 1959, Manop, col., light (1 specimen USNM); Bangkok, 28 II 1971, P. & P. Spangler (10 specimens USNM). **Chiang Mai Province**: Amphur Hot, 18°09.930'N, 98°13.496'E, 870 m, pond, 18 V 2004, L-695 (69 specimens UMC); Chiang Mai, 10 III, 1952 m D. C. & E. B. Thurman (2 specimens SEMC). **Chaiyaphum Province**: Tad Tone National Park, Namtok Tad Tone, 15°58.796'N, 102°02.079'E, 210 m, 29 IV 2004, A. Vitheepradit, L-650 (1 specimens UMC); Amphur Chum Pae, 16°34.585'N, 102°01.668'E, 217 m, 3 V 2004, A. Vitheepradit, L-660, (4 specimens UMC). **Chumphon Province**: Amphur Sa Wi, Ban Kron, 10°14.542'N, 99°05.555'E, 6 m, 30 V 2004, L-729, Vitheepradit & Prommi (3 specimens UMC); Amphur Lamae, Ban Suan Som Boon, 09°43.311'N, 99°06.208'E, 13 m, 20 VI 2006, L-964, Vitheepradit & Prommi (1 specimen UMC). **Khon Kaen Province**: Amphur Chum Pae, Tumbon Noan Udom, 16°31.267'N, 102°11.323'E, 217 m, 15 IV 2009, vegetated pond, L-1039 (25 specimens UMC, 1 specimen SHSU); Khon Kaen, Khon Kaen, Bung Kaen Nakhon, 19 II 1994, William D. Shepard (1 specimen USNM); Khon Kaen City, 21 V 1954, R. E. Elbel (1 specimens USNM). **Krabi Province**: Amphur Mueang, Noppharat Thara Beach, 08°02.625'N, 98°48.517'E, 13 m, 8 I 2006, L-902 (8 specimens UMC). **Loei Province**: Amphur Nong Hin, Tumbon Nong Hin, 17°05.658'N, 101°49.193'E, 328 m, 4 V 2004, L-662, A. Vitheepradit (2 specimens UMC); Amphur Nong Hin, Ban Huay Deur, 17°05.804'N, 101°49.823'E, 316 m, 4 V 2004, L-663, A. Vitheepradit (1 specimens UMC). **Mae Hong Son Province**: Tam Pla Resort at Tam Pla River, 19°25'34.2"N, 97°59'16.7"E, 21 X 1997, Wolfgang G. Ulrich (1 specimen USNM). **Nakhon Ratchasima Province**: Nakhon Ratchasima, 60 km S of Sakaerat Experimental Station, 30–600 m, 14°30'N, 101°55'E, 2–4 III 1972, P. & P. Spangler (18 specimens USNM). **Nakhon Sawan Province**: Tumbon Mae Poen, 15°39.054'N, 99°28.727'E, 119 m, 23 V 2004, L-708, Vitheepradit & Prommi (11 specimens UMC); Kamphaeng San, Kasetsart University Campus, 14°00.790'N, 99°59.359'E, 15 I 2012, L-1321, among *Azolla pinnata* in pond, A. Vitheepradit, T. O. Prommi & R. W. Sites (1 specimen SHSU). **Phetchaburi Province**: Amphur Nong Ya Plong, Tumbon Nong Ya Plong, 13°09'N, 99°41'E, 69 m, 15 V 2003, L-533, Vitheepradit & Ferro (10 specimens UMC); Amphur Tha Yang, Mae Nam Phetchaburi, on HWY 3499, 12°55'N, 99°51'E, 39 m, 19 IV 2002, Vitheepradit & Kirawanich, L-354 (1 specimen). **Phuket Province**: mtn. stream 3 III 1968, B. A. Harrison (15 specimens). **Prachuap Khiri Khan Province**: Amphur Hua Hin, Ban Nong Yai Oum, 12°35'N, 99°46'E, 83 m, 16 V 2003, Vitheepradit & Ferro, L-539 (12 specimens UMC); Amphur Thap Sakae, Ban Huay Yang, 11°36'N, 99°38'E, 25 m, 18 V 2003, Vitheepradit & Ferro, L-543 (2 specimens UMC). **Sakon Nakhon Province**: Sakonnakhora (sic) city reservoir, 3 II 1952, M. E. Griffith (16 specimens SEMC). **Sara Buri Province**: Amphur Sao Hal (2.7 km west), 14°33'N, 100°49'E, 11 III 1971, P. & P. Spangler (1 specimen USNM). **Songkhla Province**: Amphur Rataphum, Tumbon Kampangphet, 07°08.030'N, 100°18.437'E, 30 m, 2 VI 2004, Vitheepradit & Prommi, L-732 (38 specimens UMC, 1 specimen SHSU); Amphur Hat Yai, Prince of Songkla University, pond near reservoir, 07°00'N, 100°30'E, 58 m, 4 V 2002, Vitheepradit & Kirawanich, L-391 (1 specimen UMC). **Surat Thani Province**: Amphur Phuphin, Tumbon Khao Kwai, 09°03.773'N, 99°14.521'E, 17 m, 16 VI 2004, Sites, Vitheepradit, & Prommi, L-771 (2 specimens SHSU); Amphur Ban Na, Ban Huay Harng, 08°50.925'N, 99°18.215'E, 24 m, pond, 20 VI 2004, Vitheepradit & Prommi, L-781 (1 specimen, UMC); Amphur Ban Na, Ban Tha Rau Tai, 08°56.567'N, 99°15.130'E, 4 m, 20 VI 2004, L-782, Vitheepradit & Prommi (1 specimen SHSU); Amphur Ban Na San, Ban Poo Pea, 08°40.490'N, 99°20.092'E, 4 m, 19 VI 2006, L-962, Vitheepradit & Prommi (36 specimens UMC, 1 specimen SHSU). **Tak Province**: Amphur Meung, Tumbon Nhong Bua Tai, 16°49'N, 99°07'E, 106 m, 9 V 2003, L-514 (50 specimens UMC, 1 specimen SHSU). **Trang Province**: Amphur Sikao, pond at Chao Mai Beach, 07°26.842'N, 99°20.647'E, 3 m, 9 I 2003, Vitheepradit & Prommi, L-907 (28 specimens UMC); Prince of Songkla University, 7°31'N, 99°35'E, 55 m, 7 VI 2003, CMU and PSU teams, L-597 (13 specimens UMC, 1 specimen SHSU). **Uthai Thani Province**: Amphur Ban Rai, Tumbon Kang Roong, 15°14.121'N, 99°41.002'E, 69 m, 24 V 2004, L-712, Vitheepradit & Prommi, (3 specimens UMC); Amphur Mueang, Tumbon Nam Serm, 15°20.886'N, 100°02.120'E, 20 m, 24 V 2004, L-713, Vitheepradit & Prommi (1 specimen UMC). **Northeast** Thailand, 15 I 1953, M. E. Griffith (90 specimens SEMC, 5 specimens SHSU).

#### Discussion.

Because of the wide geographic distribution and variable characters, it is possible that what has previously been considered to be *P. frontalis* may include more than one species. Previously, it has not been possible to differentiate between species variation and species boundaries. However, after viewing hundreds of specimens, it appears that there are some reliable characters as long as there is a series of specimens in the sample to account for the variation. Lundblad (1933) noted that this is a species with variable characters, which is supported by the findings of this study. If a single specimen is used for identification, there may be some uncertainty in obtaining an accurate identification. The most apparent diagnostic character of *P. frontalis* is the facial marking found in most specimens, consisting of five dark marks, one vertical stripe on the center of the face and two pairs of horizontal stripes (Fig. 1C). Nearly 90% of specimens examined had these markings (although with variation in how dark these markings appear); thus, with a series of specimens, the species as now defined is readily identified. The current data did not include the type specimens (because they were never designated); however, they did match this diagnostic characteristic given in the original description. *Paraplea brunni* (Kirkaldy) and *P. halei* (Lundblad) also commonly have facial markings but they are usually restricted to the center vertical stripe and are not known to have the full component in the pattern shown in Fig. 1C. The scutellum in most specimens of *P. frontalis* is a contrasting lighter color and is often golden-orange. The keel of these specimens is also usually diagnostic, having well-defined teeth on the abdominal segments that are relatively longer than in other species, but there is variation in this character. Some specimens have smaller teeth, which could have resulted from wear, and the shape of these teeth can vary from being relatively straight to curved. The drawings of the keel by Lundblad (1933) of specimens from Indonesia had smaller teeth on the abdominal keel than did the majority of those examined in this study, but without examining the Indonesian specimens, it is uncertain if this is typical of specimens from these islands. As with most species of Pleidae, the ovipositor and subgenital plates of both sexes are diagnostic of the species. The ovipositor of *P. frontalis* most commonly has the teeth as shown in Fig. 3, but some specimens appear to be lacking inner teeth. This could be slight intraspecific variation or possibly interspecific differences if *P. frontalis* proves to be a species complex.

### Paraplea lateromaculata

Taxon classificationAnimaliaHemipteraPleidae

Cook sp. nov.

8A253831-5060-53F5-91C9-E10E39E88910

http://zoobank.org/377ED572-8BF9-4EAA-850B-7852358A97D3

**Figure 6.**
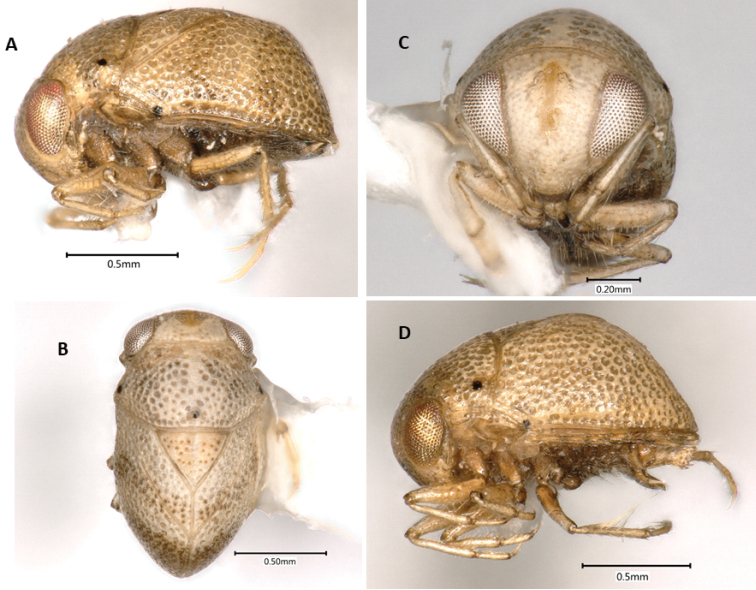
*Paraplea lateromaculata* sp. nov. specimens from Thailand. **A** mele in lateral view with typical coloration. **B** dorsal view of a light colored morph that shows dark banding and honeycombing. **C** frontal view of specimen in B. **D** female showing an alternatively colored form in lateral view without dark bands.

**Figures 7–8.**
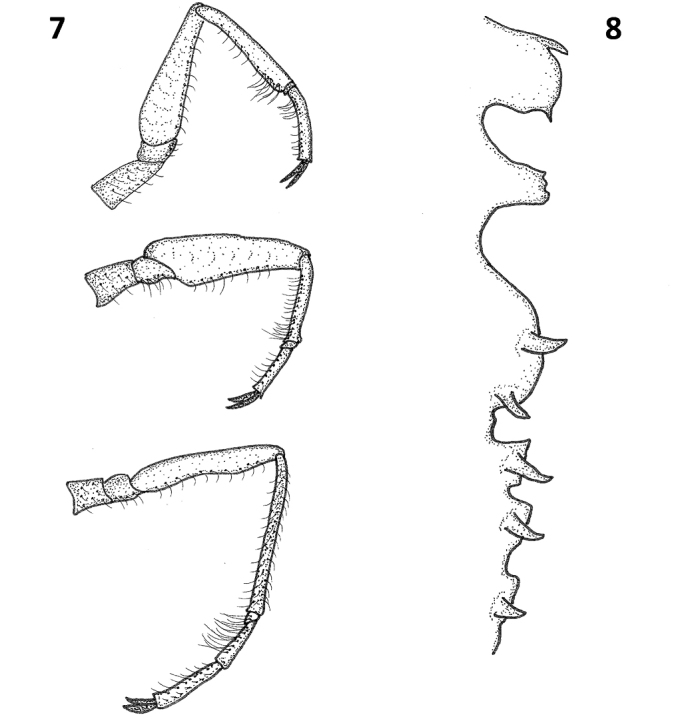
*Paraplea lateromaculata* sp. nov. **7** prothoracic leg above, mesothoracic leg in the middle and metathoracic leg below. **8** profile of the ventral keel with the anterior end (thoracic) to the top and ventral to the left.

**Figure 9.**
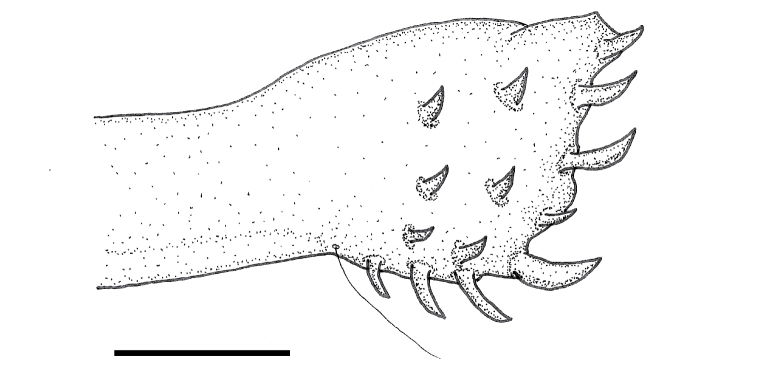
Ovipositor of *Paraplea lateromaculata* sp. nov. Scale bar: 0.05 mm.

**Figures 10–11.**
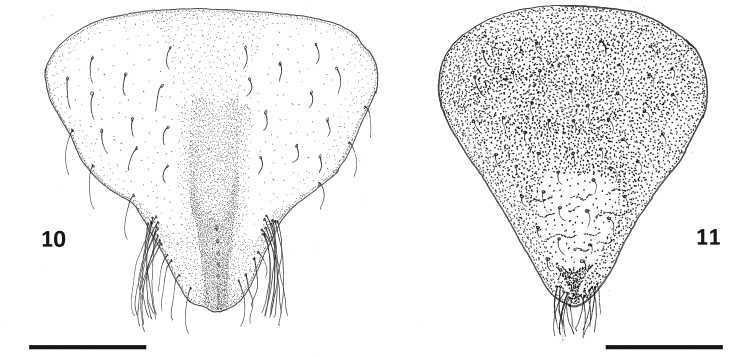
Genital plates of *Paraplea lateromaculata* sp. nov. **10** female, **11** mele. Scale bar: 0.1 mm.

Figures 6 , 7–8 , 9 , 10–11 , 24B

#### Description.

All measurements are in millimeters and were taken from 224 adult specimens from throughout Thailand as reported in the distribution portion of this paper. Specimens used for this description are deposited at SEMC, SHSU, and UMC.

***Body size*.** Total length, 1.21–1.58 (average 1.42) (Fig. 6A, B, D); two specimens not included in this range unusually large, 2.35 and 2.52, but consistent in all other morphological characters with this species.

***Color*.** Color may vary slightly among individuals (Fig. 6A, B, D). Base color of body most often golden-tan with some darker brown markings. A few specimens exhibit a weak banding pattern of the hemelytra (Fig. 6A), banding more pronounced in small percentage of individuals that are more lightly colored (Fig. 6B); many with some honeycombing (Fig. 6B, D); small percentage of lightly colored specimens with red patches on vertex of head. Nearly all specimens with three dark spots on pronotum, two near posterolateral region and one at dorsal middle posterior. Distinctive dark spot on hemelytra above metacoxa (Fig. 6A, D).

***Head*.** Head (Fig. 6C) generally light-brown to cream-colored, often with darker markings between eyes. Face and vertex often with a distinctive vertical bar (Fig. 6C), although sometimes missing or not distinctively bar-shaped. Antenna three-segmented, usually hidden from view below eye (extended and visible in Fig. 1A). Head size similar among Thai specimens, head width (excluding two unusually large specimens noted above) at widest point including eyes 0.62–074 (average 0.67), head width at narrowest point between eyes, 0.29–0.39 (average 0.35), OI 45–55 (average 50).

***Pronotum*.** Base color usually light-tan but ranging between nearly white to brown, usually with lighter colored honeycombing apparent; most specimens have three distinct dark spots, one near posterolateral edge and one near the central posterior margin of pronotum (Fig. 6B); most have visible central vertical band without punctures, at least at anterior end (Fig. 6B, C); slight bulge present toward lateral posterior; punctures present, ~ 0.02; if honeycombing present, punctures located between honeycomb bars (Fig. 6B, D); pronotum width 0.58–0.84 (average 0.74); pronotum length 0.29–0.63 (average=0.42); PI 38–69 (average 57).

***Wings*.** Complete to posterior; punctures evenly dispersed with only small distance between punctures, not in rows (0.02 – 0.03 in diameter) (Fig. 6A, D); underlying honeycomb structure sometimes present; claval suture present in most (Fig. 6A) but absent in some (Fig. 6D); scutellum with distinct punctures, usually darkened in center (Fig. 6B), more widely spaced than other punctures, punctures not in apparent order; scutellum base color similar to hemelytra, almost white to golden-brown; lateral view shows distinct black spot near margin (Fig. 6A, D), resembling spots on prothorax; darker vertical band on some specimens (Fig. 6B); shape of hemelytra ranging from rectangular (Fig. 6A) to having a dorsal bulge (Fig. 6D); scutellum slightly wider than long but often almost triangular (Fig. 6B), scutellum length 0.21 – 0.43 (average 0.30); scutellum width 0.28–0.48 (average .36); SI 106–148 (average 120). Hind wings membranous, fully-developed, completely concealed by hemelytra.

***Legs*.** Shape of legs as in Fig. 7. Typical leg measurements: prothoracic leg coxa 0.14, trochanter 0.06, femur 0.37, tibia 0.30, first tarsomere 0.02, second tarsomere 0.15,pretarsal claw 0.10; mesothoracic leg coxa 0.14, trochanter 0.06, femur 0.37, tibia 0.21, first tarsomere 0.02, second tarsomere 0.12, pretarsal claw 0.09; metathoracic leg coxa 0.13, trochanter 0.06, femur 0.40, tibia 0.40, first tarsomere 0.05, second tarsomere 0.17, third tarsomere 0.17, pretarsal claw 0.10; several long hairs along ventral side of trochanter, femur, tibia and tarsus, especially at base of hind tarsus where some hairs reach 0.25 (Fig. 7).

***Median ventral keel*.** Thoracic portions distinct from each other, prosternal keel somewhat rectangular with small teeth at anterior and posterior edges; mesosternal keel small but distinctly squared in profile, slightly serrated; metathoracic keel segment somewhat rounded with prominent teeth. Abdominal keel on segments I-IV with distinct teeth, segment I appears fused to metathoracic keel. Figure of typical specimen in Fig. 8.

***Characters of female*.** Ovipositor roughly rectangular but apical side slightly wider (gonapophysis 1) at end of fused rectangular shaft (gonapophysis 2) (Fig. 9); five distinct teeth along posterior border (apical row), although fourth tooth is smaller and somewhat recessed. In some specimens this small tooth appears to be missing; three teeth on ventral border, decreasing in size posteriorly; two rows of three teeth each away from apex, three primary and three secondary, but usually no tertiary teeth; one long hair on ventral side where triangular apex of gonapophysis 1 and basal rectangle of gonapophysis 2 meet, however this hair is sometimes inconspicuous except at high magnification; subgenital plate as in Fig. 10; slightly wider than long; width ~ 0.28, length ~ 0.25; relatively smooth but with hairs emerging from shallow pits; tufts of relatively long hairs on each side near apex; slightly shorter hairs spaced throughout genital plate darker v-shaped prominence in center, near apex and extending 2/3 to posterior border.

***Characters of male*.** Aedeagus bulbous and somewhat asymmetrical in typical fashion of family; operculum (subgenital plate) generally triangular, slightly longer than wide (Fig. 11), width ~ 0.23, length ~ 0.25, lightly rugulose in center before apex but otherwise smooth to granular, with short hairs throughout, several longer hairs at apex.

#### Distribution.

*Paraplea lateromaculata* is found throughout most of peninsular Thailand, and few specimens have also been collected in eastern Thailand (Fig. 24B). Specimens observed include a single specimen collected from Singapore.

#### Type material examined.

***Holotype*** female, Thailand: **Krabi Province**, Amphur Nuea Khlong, Tumbon Klong Kanarn, Ban Klong Kanarn, pond 8°01.045'N, 99°00.450'E, 37 m, 8 I 2006, Vitheepradit and Prommi, L-903 (UMC). ***Paratypes*** (38 TOTAL): Singapore: Federated Malay States, 31 X 1955, Marshall Laird (1 paratype SEMC). Thailand: **Chumphon Province**: Amphur Se Wi, Ban Kron, 10°17.390'N, 99°05.464'E, 5 m, 30 V 2004, Vitheepradit and Prommi, L-728 (3 paratypes UMC, 1 paratype SHSU); Amphur Sa Wi, Ban Kron, pond, 10°14.542'N, 99°05.555'E, 6 m, 30 V 2004, Vitheepradit and Prommi, L-729 (5 paratypes UMC); Amphur Lamae, Ban Suan Som Boon, 09°43.311'N, 99°06.208'E, 20 VI 2006, Vitheepradit and Prommi, L-964 (4 paratypes UMC, 1 paratype SHSU). **Kalasin Province**: Phu Pan National Park, Lahm Huay Noi, 8 km S of Ban Kahm Perm, vegetated margins of river, 7 VI 1998, Vitheepradit, Sites and Simpson, L-162 (1 paratype UMC). **Loei Province**: Amphur Phukraduna, pond 1 km W of intersection of Hwy 201 and 2019, 21 VI 1998, Vitheepradit and Sawangsak, L-201 (1 paratype UMC). **Nong Bua Lamphu Province**: Phu Kao-Phu Pan Kham National Park, Namtok Tad Fah Waterfall, 16°55.259'N, 102°27.659'E, 201 m, 10 V 2004, Prommi and Vitheepradit, L-674 (2 paratypes UMC). **Phatthalung Province**: Praiwan Waterfall, 3 km W of Ban Phut, pond with vegetation, 11 VII 1997, Sites and Permkam, L-135 (2 paratypes UMC). **Phetchaburi Province**: Amphur Tha Yang, Ban Yang Chum, stream, 12°47'N, 99°40'E, 46 m, 15 V 2003, Vitheepradit and Ferro, L-536 (3 paratypes UMC, 1 paratype SHSU). **Prachuap Khiri Khan Province**: Amphur Kui Buri Forest, Forest Plantation Station, 12°04'N, 99°37'E, 17 V 2003, 117 m, Ferro and Vitheepradit, L-540 (1 paratype UMC). **Songkhla Province**: Amphur Ratephum, Tumbon Kampangphet, 7°08.030'N, 100°18.437'E, 30 m, 2 VI 2004, 30 m, Vitheepradit and Prommi, L-732 (1 paratype UMC). **Trang Province**: Amphur Sikao, Tumbon Mai Fard, Ban Klong Maeng, pond, 7°30'N, 99°20'E, 6 m, 10 VIII 2005, Vitheepradit, Simpson and Prommi, L-868 (4 paratypes UMC, 2 paratypes SHSU); Amphur Sikao, Tumbon Mai Fard, Ban Klong Maeng, pond, 7°30.170'N, 99°20.541'E, 6 m, 10 I 2006, Vitheepradit and Prommi, L-908 (1 paratype UMC). **Northeast** Thailand, 15 I 1953 M. E. Griffith (4 paratypes SEMC, 1 paratype SHSU).

#### Additional material examined.

Thailand: **Chumphon Province**: Amphur Lamae, Ban Suan Som Boon, 09°43.311'N, 99°06.208'E, 13 m, 20 VI 2006, Vitheepradit and Prommi, L-964 (7 specimens UMC); Amphur Sa Wi, Ban Kron, 10°17.390'N, 99°05.464'E, 5 m, 30 V 2004, Vitheepradit and Prommi L-728 (2 specimens UMC); Amphur Sa Wi, Ban Kron, pond, 10°14.542'N, 99°05.555'E, 6 m, 30 V 2004, Vitheepradit and Prommi, L-729 (5 specimens UMC, 1 specimen SHSU). **Kalasin Province**: Phu Pan National Park, black light at Park Headquarters near pond; 7 VI 1998, Sites, Simpson, and Vitheepradit, L-166 (42 specimens UMC, 1 specimen SHSU); Phu Pan National Park, Lahm Huay Noi, 8 miles S of Ban Kahm Perm, vegetated margins of river, 7 VI 1998, Vitheepradit, Sites and Simpson, L-162 (3 specimens UMC). **Kanchanaburi Province**: Thong Pha Phum, Reforestation Station, black light, 14°39'N, 98°35'E, 211 m, 11 IV 2002, UMC & CMU teams, L-333 (1 specimen UMC). **Krabi Province**: Amphur Nuea Khlong, Tumbon Klong Kanarn, Ban Klong Kanarn, pond 8°01'N, 99°00'E, 37 m, 8 I 2006, Vitheepradit and Prommi, L-903 (2 specimens UMC). **Loei Province**: Amphur Phukradung, pond 2 km W of intersection of Hwy 201 and 2019, 21 VI 1998, Vitheepradit and Sawangsak, L-201 (6 specimens UMC). **Nakhon Si Thammarat Province**: 6 km N of Amphur Chulabhorn on Hwy 41, pond, 8°07.625'N, 99°51.540'E, 31 m, 8 VI 2004, Vitheepradit and Prommi, L-756 (1 specimen UMC); Amphur Chulabhorn, Tumbon Na Moh Boon, 8°01.664'N, 98°53.763'E, pond, 20 m, 4 VI 2004, Vitheepradit and Prommi, L-741 (1 specimen UMC); Nopphitam Khlong Yod Leung, stream, 8°38'N, 99°44'E, 78 m, 26 V 2005, Vitheepradit and Prommi, L-800 (1 specimen UMC). **Nong Bua Lamphu Province**: Phu Kao-Phu Pan Kham National Park, Namtok Tad Fah, 16°55.259'N, 102°27.659'E, 201 m, 10 V 2004, Prommi and Vitheepradit, L-674 (1 specimen UMC). **Phatthalung Province**: Praiwan Waterfall, 3 km W of Ban Phut, pond with vegetation, 11 VII 1997, Sites and Permkam, L-135 (2 specimens UMC); Amphur Phayom, Ban Pa Phayom, pond, 7°50'N, 99°56'E, 19 m, 31 V 2003, Vitheepradit and Ferro, L-584 (3 specimens UMC). **Phetchaburi Province**: Amphur Tha Yang, Ban Yang Chum, stream, 12°47'N, 99°40'E, 46 m, 15 V 2003, Vitheepradit and Ferro, L-536 (8 specimens UMC). **Songkhla Province**: Amphur Rataphum, Tumbon Kampangphet, 7°08.030'N, 100°18.437'E, 30 m, 2 VI 2004, Vitheepradit and Prommi, L-732 (3 specimens UMC); Hat Yai, Prince of Songkla University campus ponds, 21 VI 2002, Sites and Permkam, L-411 (4 specimens UMC); Prince of Songkla University, pond near reservoir, 7°00'N, 100°30'E, 58 m, 8 VI 2005, Prommi, Sites and Vitheepradit, L-834 (7 specimens UMC). **Surat Thani Province**: Amphur Ban Na, Ban Tha Rau Tai, pond, 8°56.567'N, 99°15.130'E, 4 m, 20 VI 2004, Vitheepradit and Prommi, L-782 (3 specimens UMC). **Trang Province**: Amphur Sikao, Tumbon Mai Fard, Ban Khlong Maeng, pond, 7°30'N, 99°20'E, 6 m, 4 June 2005, Sites, Vitheepradit and Prommi, L-831 (1 specimen UMC); Amphur Sikao, Tumbon Mai Fard, Ban Khlong Maeng, pond, 7°30'N, 99°20'E, 6 m, 10 I 2006, Vitheepradit and Prommi, L-908 (44 specimens UMC); Amphur Sikao, Tumbon Mai Fard, Ban Khlong Maeng, pond, 7°30'N, 99°20'E, 6 m, 14 VI 2006,Vitheepradit, Sites and Prommi, L-955 (25 specimens UMC). **Northeast** Thailand, 15 I 1953, M. E. Griffith (30 specimens SEMC).

#### Etymology.

The specific epithet combines two Latin roots, *latero*- meaning the side and –*macula* meaning spot. Thus, the name refers the distinct dark spot on the lateral side of the hemelytra. This spot is similar to the dorsal pronotal spots found in this species and *P. liturata*.

#### Discussion.

In general appearance, *P. lateromaculata* sp. nov. could be misidentified as *P. liturata* that is missing two of its dark pronotal spots. However, several consistent characters separate these species. The most obvious of these characters is that *P. lateromaculata* sp. nov. has one dark spot on each side of the hemelytra, anteriorly near the costal margin, which is absent in *P. frontalis*. The ovipositors of these species are quite different (compare Fig. 3 with Fig. 9), and diagnostic characters of the ventral keel and genital plates also differ between these species. *Paraplea lateromaculata* sp. nov. is much smaller, none of which had body lengths reaching a length of 1.60 compared to the smallest measured *P. frontalis* at 1.89.

*Paraplea lateromaculata* sp. nov. can be differentiated from *P. melanodera* sp. nov. by their colored markings. More specifically, *Paraplea lateromaculata* sp. nov. has the distinctive black spots whereas *P. melanodera* sp. nov. has no black spots but has a black band at the posterior margin of the head. Although the size is similar between *P. lateromaculata* sp. nov. and *P. melanodera* sp. nov., recognizable differences exist in other characters as listed in their respective descriptions. *Paraplea lateromaculata* sp. nov. often has three pronotal black spots on the prothorax as is found in less than 10% of *P. liturata*; however, *P. liturata* has never been observed to have the black spots on the hemelytral costal margin as is seen in all specimens of *P. lateromaculata* sp. nov.

### Paraplea liturata

Taxon classificationAnimaliaHemipteraPleidae

(Fieber, 1844)

5AB41CA0-4AE0-5C10-9CFB-D972E444FCF3

**Figure 12.**
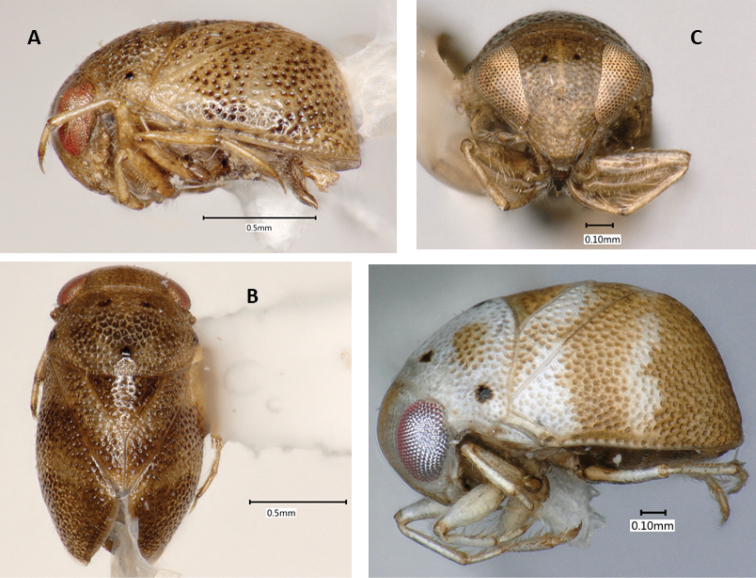
*Paraplea liturata* specimens from Thailand. **A** female in lateral view with typical coloration, **B** dorsal view in that shows dark banding and honeycombing. **C** frontal view of specimen in B. **D** mele showing an alternatively colored form in lateral view.

**Figures 13, 14.**
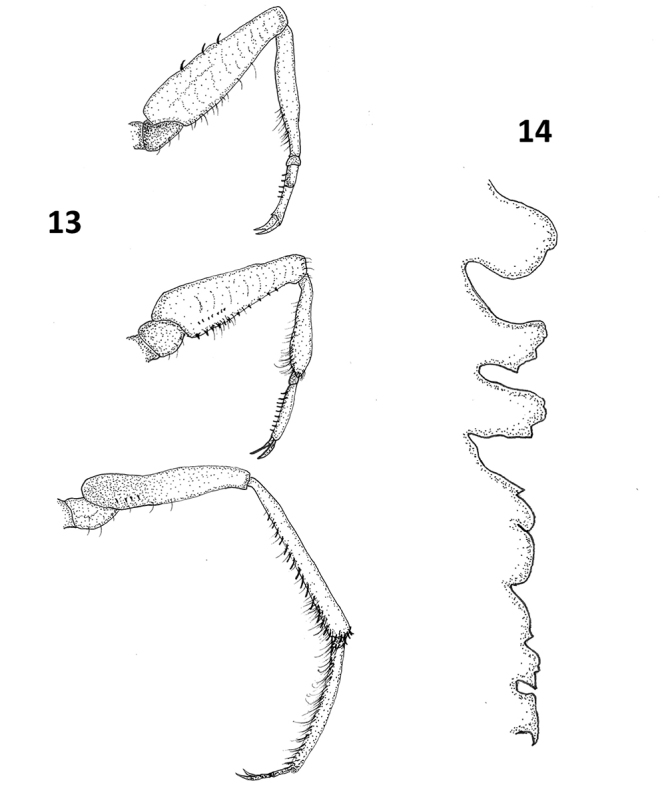
*Paraplea liturata.***13** prothoracic leg above, mesothoracic leg in the middle and metathoracic leg below. **14** profile of the ventral keel with the anterior end (thoracic) to the top and ventral to the left.

**Figure 15.**
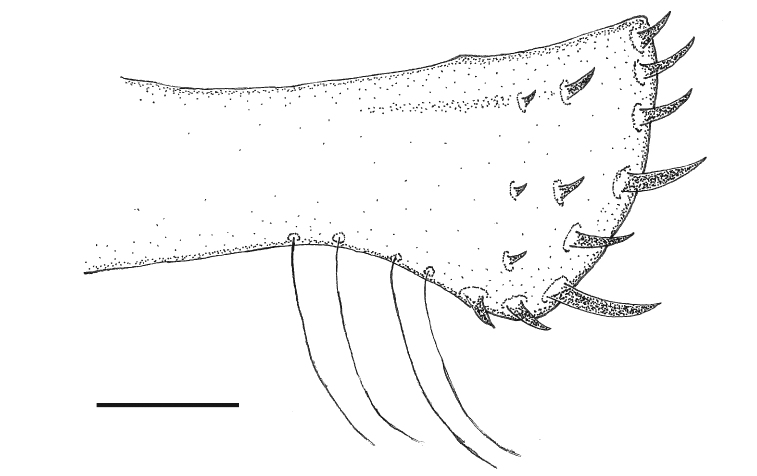
Ovipositor of *Paraplea liturata*. Scale bar: 0.05 mm.

**Figure 16, 17.**
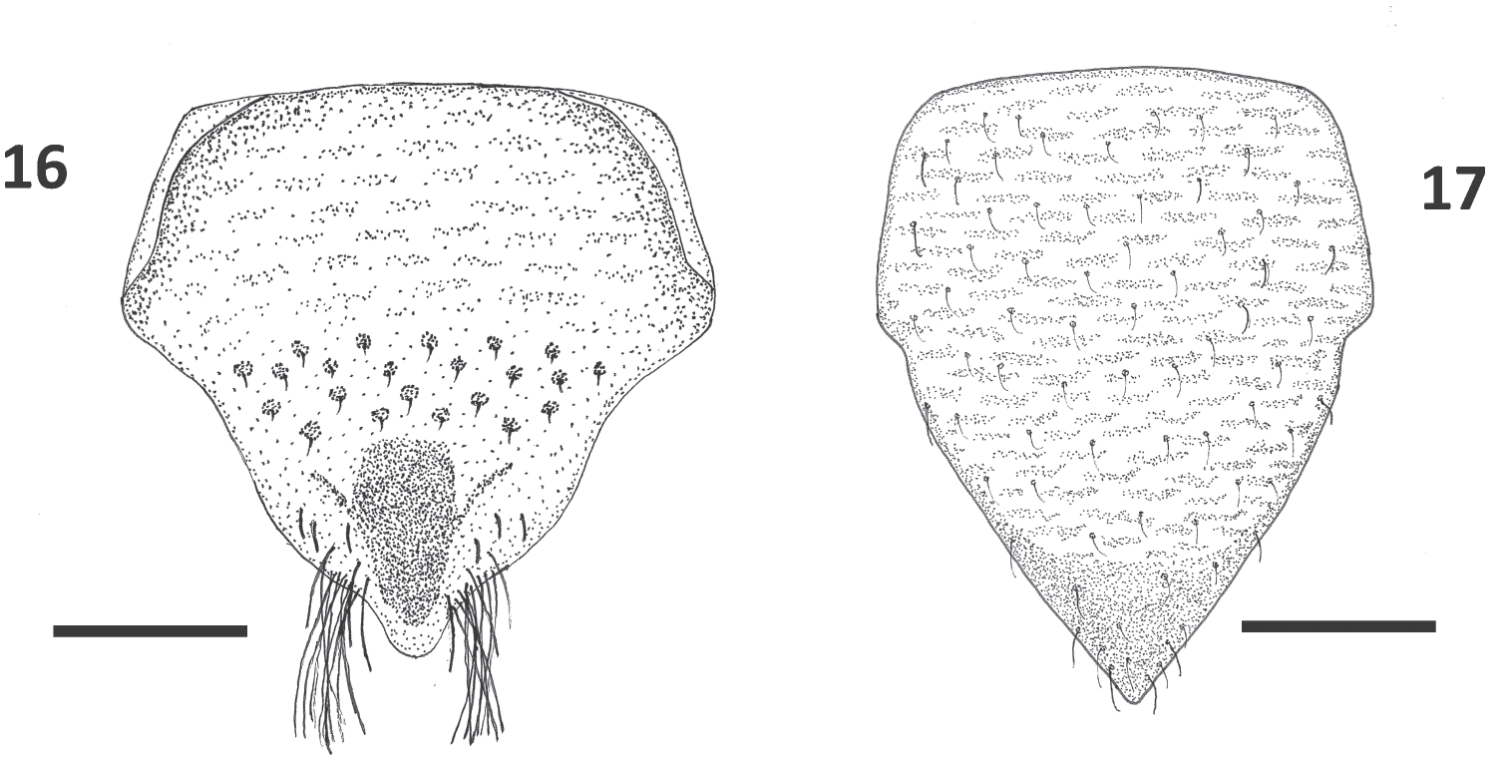
Genital plates of *Paraplea liturata*. **16** female **17** mele. Scale bar: 0.1 mm.

Figures 12 , 13, 14 , 15 , 16–17 , 24C

= Ploa liturata Fieber, 1844: Entomol. Monogr. p. 19.
= Plea liturata: (Kirkaldy, 1904): Wien. Entomol. Zeit. 23: 129.
= Plea metiadusa Distant, 1910: Fauna Brit. India 5: 337.
= Plea rufonotata Distant, 1914: Rech. Sci.N.-Calédonie 2: 387.
= Plea quinquenota Paiva, 1918: Rec. India. Mus. 14: 29.
= Plea fasciata Horváth, 1918: Ann. Mus. Nat. Hung. 16: 144.
Plea (Paraplea) liturata : Esaki and China 1928: Rev. Esp. Entomol. 4: 166 (subgenus description).
Paraplea liturata : Drake and Maldonado-Capriles 1956 (elevation to genus) 51: 53.

#### Remarks.

*Paraplea liturata* shares some of the same taxonomic history as *P. frontalis* since both were described in the same paper. *Paraplea liturata* was described as *Ploa liturata* by Fieber (1844) for specimens collected in the East Indies. This original description was not only brief but did not capture an important set of markings that is commonly present on specimens of *P. liturata*, even though the description relied almost completely on coloration. Fieber noted the typical two dashes between the eyes and the pair of lateral and single midline spots on the posterior margin of the pronotum. However, two spots on the anterior part of the pronotum were not listed in the description nor on his plate. The figure does show the hemelytral banding that is common in many specimens. Although types were not designated by Fieber, subsequent researchers were able to associate the description with many specimens of this common species, although none of these were those used by Fieber. Placement of this species into the genus *Paraplea* followed the same sequence as reported for *P. frontalis*. Kirkaldy (1904) moved this species into the genus *Plea* but made no comments on the species. Distant (1906) translated Fieber’s description but did not further report on this species. Distant (1910) described *Plea metiadusa* from Calcutta, India and reported that it had no maculations, but this species was still determined to be a variant of *P. liturata* by Lundblad (1933). In doing so, Lundblad (1933) commented on the variability of the pronotal spots that are most commonly five in number but can range from none to seven. Distant (1914) also described *Plea rufonotata* but did not associate it with *P. liturata*. The description of *P. rufonotata* from New Caledonia was the first time that a pronotum with five spots was described, which now appears to be the most common state for *P. liturata*. Again, Lundblad (1933) made the association of these species as being synonyms. Horváth (1918) described *Plea fasciata* from specimens from Batavia, Java (Indonesia) but this was considered a strongly-colored example of *P. liturata* (Lundblad 1933). *Plea quinquenota* (Paiva 1918) was described from a single specimen from Inlé Lake, Yawnghe State (now in Myanmar) and was not examined by Lundblad (1933), but was still synonymized with *P. liturata* based on the illustration of the new species perfectly matching the typical form of *P. liturata*. Along with the taxonomic clarifications, Lundblad (1933) also redescribed the species. Below is a supplemental description of the species, incorporating specimens from Thailand.

***Holotype*.** None designated.

#### Type locality.

Fieber (1844) reported that the description was made from specimens collected by Dr. Helfer in the East Indies but the exact location in South or Southeast Asia is unknown.

#### Supplementary description.

All measurements are in millimeters and were taken from 221 adult specimens from throughout Thailand as reported in the distribution portion of this paper. Specimens used for this description are deposited at UMC and SHSU.

***Body size*.** Total length, 1.27–1.68 (average 1.48) in Thailand specimens. Fieber (1844) reported total length of “approximately 2 mm.” Distant (1910) gave the size of his later synonymized *P. metiadusa* from India and his later synonymized *P. rufonotata* from Caledonia as 2. The specimen used to describe the later synonymized *P. quinquenotata* was listed as 1.5 (Paiva 1918). Anderson and Weir (2004) reported a length of 1.8–2.0 for this species in their key and reported it from Northern Territory and Western Australia.

***Color*.** Color may be quite variable among individuals within a population. Base color of body ranges from tan (Fig. 12A, B) to white (Fig. 12D). Darker bands on the sides of the hemelytra are common but not observed in all individuals. When present, bands are darker than the base color, ranging from light orange-brown (Fig. 12D) to tan (Fig. 12A) to brown (Fig. 12B). Punctures are sometimes a darker shade (Fig. 12A). Honeycombing matches the base color. Most specimens have five characteristic dark spots on the pronotum (Fig. 12B); two on the anteromedial portion (Fig. 12 B), two on the posterolateral region (one on each side) (Fig. 12B, D), and one on the posteromedial region (Fig. 12B, D). Conversely, these spots are sometimes faint or absent in some individuals and at some locations.

***Head*.** Head (Fig. 12C) colored with base body coloration, ranging from white to brown. Many specimens with a vertical light-colored bar between eyes. If present, bar can be thin (Fig. 12C) to wider, sometimes occupying nearly a third of width between eyes. Two dark spots common on face, one on each side between central bar and eyes (Fig. 12C). Eyes in dried specimens range from red to gold. Mouthparts usually darker that the rest of head. Antenna three-segmented, usually hidden from view below eye. Head size similar among Thailand specimens, head width at widest point including eyes 0.62–0.79 (average 0.72), head width at narrowest point between eyes, 0.31–0.43 (average 0.40), OI 47–58 (average 53).

***Pronotum*** (Fig. 12B). Base color ranging from white to light brown and honeycombing apparent in most specimens; most specimens with five dark spots on pronotum (91% of Thailand specimens with five spots, ~ 9% with three spots and lacking anterior pair, less than 1% with no spots); a shallow puncture in center of each cell of honeycomb and under high magnification a minute hair can usually be seen coming from each pore; with slight bulge posteriorly, wider than long, width 0.66–0.92 (average 0.79); pronotum length 0.33–0.57 (average 0.46); PI 39–68 (average 58).

***Wings*.** Complete to posterior; punctures equally spaced but not generally in rows (0.03 in diameter) (Fig. 12A, D); underlying honeycomb structure usually present; claval suture distinct, complete; scutellum with punctures smaller (0.01 in diameter), more widely spaced than other punctures, scutellum base color often golden but sometimes dark brown and often darker than hemelytra; honeycombing absent from scutellum, making it appear somewhat transparent; lateral view shows distinct horizontal dorsal profile and near vertical aspects posterior profile with a transition of nearly 90° (Fig. 12A, C); scutellum slightly wider than long (Fig. 12B), length 0.28–0.43 (average 0.35); width 0.31–0.50 (average 0.40); SI 103–139 (average 116). Hind wings membranous, fully developed, completely concealed by hemelytra.

***Legs*.** Legs with numerous hairs and small spines (Fig. 13), hairs prevalent on apical half of prothoracic and mesothoracic tibiae, without numerous long hairs as found on tibia and tarsus of many *Paraplea*. Typical leg measurements: prothoracic leg coxa 0.05, trochanter 0.09, femur 0.42, tibia 0.35, first tarsomere 0.04, second tarsomere 0.09,pretarsal claw 0.08; mesothoracic leg coxa 0.06, trochanter 0.10, femur 0.41, tibia 0.28, first tarsomere 0.03, second tarsomere 0.13, pretarsal claw 0.08; metathoracic leg coxa 0.05, trochanter 0.10, femur 0.39, tibia 0.49, first tarsomere 0.02, second tarsomere 0.13, third tarsomere 0.18, pretarsal claw 0.11.

***Median ventral keel*.** Thoracic portions distinctly separate, prothoracic keel generally rounded, two posterior thoracic segments serrated; abdominal keel with first two segments fused, teeth more pronounced posteriorly (Fig. 15). Several minor differences between Thailand specimens reported here and those reported by Lundblad (1933: fig. 42C–E).

***Characters of female*.** Ovipositor most commonly as in Fig. 15. Ovipositor roughly rectangular in shape but with apical gonapophysis 1 slightly wider; five distinct teeth along posterior border (apical row) plus two teeth on ventral border (ventral 1 and 2); two rows of small teeth away from apex, three primary, three secondary, and occasionally one tertiary (not shown in Fig. 15); bottom secondary tooth larger and extends slightly beyond end of ovipositor, making it sometimes appear as being along posterior margin; three to five long hairs on ventral side of region where gonapophyses 1 and 2 meet; Subgenital plate slightly longer than wide (Fig. 16), length ~ 0.30, width ~ 0.26, lightly rugose in basal half followed apically by a series of pits, dark region in center near apex, pair of tufted hairs on each side near apex.

***Characters of male*.** Aedeagus bulbous and somewhat asymmetrical in typical fashion of family; operculum (subgenital plate) as in Fig. 17, most of surface slightly rugose, longer than wide, length ~ 0.27, width ~ 0.20.

#### Distribution.

In Thailand, *Paraplea liturata* appears to be mostly a peninsular species on the southwest side of the country, although there are two records of it in the eastern region of Thailand; one in Sakon Nakhon Province, which was reported by Chen et al. (2006), and one by RWS and AV in a pond in Ubon Ratchathani Province (Fig. 24C). It is also known from Australia (Anderson and Weir 2004), India (Paiva 1918. Lundblad 1933), Indonesia (Lundblad 1933, Nieser and Chen 1999), Malaysia (Fernando and Cheng 1974), Myanmar (Paiva 1918), New Caledonia (Lundblad 1933), Philippines (Lundblad 1933, Nieser and Chen 1999), and Taiwan (Mitamura et al. 2018).

#### Material examined.

**Chai Nat Province**: Amphur Neon, Kham, Ban Wang Kor Hai, 14°57.934'N, 99°50.668'E, 24 V 2004, Vitheepradit & Prommi, L-710 (1 specimen UMC). **Chumphon Province**: Amphur Sa Wi, Tumbon Kron, 10°17.499'N, 99°05.530'E, 21 VI 2006, Vitheepradit & Prommi, L-967 (4 specimens UMC). **Kalasin Province**: Phu Pan National Park, 7 VI 1998, Sites, Simpson & Vitheepradit, L-165 (3 specimens UMC, 1 specimen SHSU). **Krabi Province**: Amphur Mueang, Klong Muang Beach, pond, 8°02.979'N, 98°45.540'E, 13 m, 8 VIII 2005, Sites, Vitheepradit, Simpson & Prommi L-862 (8 specimens UMC); Amphur Mueang, Nopphorat Thara Beach, pond, 8°02.625'N, 98°48.517'E, 8 V 2005, Sites, Vitheepradit & Prommi, L-805 (1 specimen UMC); Amphur Nuea Khlong, Tumbon, Nuea Khlong, Ban Paga Sai, pond, 8°02.619'N, 99°01.144'E, 27 m, 8 VIII 2005, Sites, Vitheepradit, Simpson & Prommi, L-864 (2 specimens UMC); Amphur Nuea Khong, Pan Paga Sai, pond, 8°02.619'N, 99°01.144'E, 27 m, 13 VI 2006, Vitheepradit & Prommi, L-951 (1 specimen UMC); Tumbon Klong Kanarn, Ban Klong Kanarn, pond, 8°01.045'N, 99°00.450'E, 37 m, 9 VIII 2005, Sites, Vitheepradit, Simpson & Prommi, L-867 (2 specimens UMC). **Phang Nga Province**: Amphur Mueang, Tumbon Na Pring, pond, 8°31.750'N, 98°32.001'E, 5 I 2006, Sites, Vitheepradit & Prommi, L-887 (2 specimens UMC); Amphur Mueang, Tumbon Na Prig, pond, 8°31.750'N, 98°32.001'E, 12 VI 2006, Sites, Vitheepradit & Prommi, L-946 (4 specimens UMC); Amphur Takua Thung, Tumbon Krasom, Ban Bang Mak, pond, 8°24.553'N, 98°27.434'E, 12 VI 2006, Sites, Vitheepradit & Prommi, L-945 (1 specimen UMC); Amphur Takua Pa, Tumbon Bang Nai Si, Ban Bang Yai, pond, 08°25.950'N, 98°23.192'E, 20 m, 8 VI 2006, Sites, Vitheepradit & Prommi, L-927 (1 specimen); Amphur Thai Mueang, Tumbon Na Teoy, Ban Bang Klee, 8°18.655'N, 98°17.552'E, 19 m, 2 VI 2005, Sites, Vitheepradit & Prommi, L-825 (8 specimens UMC); Amphur Thai Mueang, Tumbon Na Teoy, Ban Bang Klee, 8°18.655'N, 98°17.552'E, 19 m, 5 I 2006, Sites, Vitheepradit & Prommi, L-886 (11 specimens UMC); Amphur Thai Mueang, Tumbon Na Teoy, Ban Bang Klee, 8°18.655'N, 98°17.552'E, 9 VI 2006, Sites, Vitheepradit & Prommi, L-935 (11 specimens UMC). **Phatthalung Province**: Amphur Tamot, Tumbon Loh Jak Kra, 7°20.244'N, 100°01.285'E, 44 m, 3 VI 2004, Vitheepradit & Prommi, L-736 (1 specimen UMC). **Phuket Province**: Amphur Thalang, Jud peat swamp, UV pan trap, 8°07.930'N, 98°18.156'E, 24 m, 7 I 2006, Sites, Vitheepradit & Prommi, L-899 (92 specimens UMC, 5 specimens SHSU); Amphur Thalang, Jig peat swamp, 8°08.772'N, 98°17.892'E, 23 m, 7 I 2006, Sites, Vitheepradit & Prommi, L-906 (44 specimens UMC, 3 specimens SHSU); Amphur Thalang, Laem Yot peat swamp, 8°08.325'N, 98°17.927'E, 28 m, 29 V 2005, Sites, Vitheepradit & Prommi, L-807 (1 specimen UMC); Amphur Thalang, Mai Khoa peat swamp, 8°07.461'N, 98°18.193'E, 19 m, 7 I 2006, Sites, Vitheepradit & Prommi, L-900 (42 specimens UMC, 1 specimen SHSU); Amphur Thalang, Tumbon Mai Khao, Moo Ha, 8°10.718'N, 98°17.611'E, 23 m, 31 V 2005, Sites, Vitheepradit & Prommi, L-816 (1 specimen SHSU); Amphur Thalang, Tumbon Mai Khao, Moo Ha, pond, 8°10.718'N, 98°17.611'E, 23 m, 5 I 2006, Sites, Vitheepradit & Prommi, L-889 (17 specimens UMC). **Songkhla Province**: Amphur Rataphum, Tumbon Kampangphet, 07°08.030'N, 100°18.437'E, 30 m, 2 VI 2004, Vitheepradit & Prommi, L-732 (9 specimens UMC). **Surathani Province**: Amphur Ban Na Ban Huay Hamg, 8°08.925'N, 99°18.215'E, 24 m, 20 VI 2004, Vitheepradit & Prommi, L-781 (3 specimens UMC); Amphur Phunphin, Tumbon Boh Rai, 8°53.866'N, 98°08.961'E, 10 m, 7 VI 2004, Vitheepradit & Prommi, L-751 (1 specimen SHSU); Amphur Ban Na, Ban Tha Rau Tai, 8°56.567'N, 99°15.130'E, 4 m, 20 VI 2004, Vitheepradit & Prommi, L-782 (3 specimens UMC). **Trang Province**: Amphur Sikao, Tumbon Mai Fard Ban, Klong Maeng, pond, 8°30.170'N, 99°20.541'E, 6 m, 10 VIII 2005, Sites, Vitheepradit, Simpson & Prommi, L-868 (2 specimens UMC, 1 specimen SHSU). **Uthai Thani Province**: Amphur Mueang, Tumbon Nam Serm, 15°20.886'N, 100°02.120'E, 24 V 2004, Vitheepradit & Prommi, L-713 (3 specimens UMC). **Ubon Ratchathani Province**: Amphur Nam Khoon, Ban Non Yang, pond, 14°28.176'N, 104°53.782'E, 190 m, 10 IV 2004, Sites & Vitheepradit, L-613 (10 specimens UMC).

#### Discussion.

There is considerable variation in *P. liturata* if this is a single species. Lundblad (1933) mentioned this variation when synonymizing *P. fasciata*, *P. metiadusa*, *P. quinquenotata*, and *P. rufonotata* with *P. liturata*. The main basis for Lundblad synonymizing these species was the similarity of the abdominal keel. The drawings in his paper (Lundblad 1933) show similarities but there are also subtle variations. The Thailand specimens are also somewhat different from those in the Lundblad drawings. These data preserve the question as to whether this is a variable species or multiple species. Several of the species Lundblad synonymized had been described as having a length of 2 mm, although he stated that this was a small species varying between 1.3 to 1.7 mm. This size range of Lundblad’s specimens coincides with that of specimens from Thailand; however, it still does not account for those described from India, New Caledonia, and Australia. The original description of *P. liturata* by Fieber (1844) listed the length imprecisely as “approximately 2 mm” and it is uncertain where in the East Indies these specimens were collected or how accurately that measurement was made. Likewise, some distinct differences in coloration and characters occur in specimens from the various regions.

A character that links all of these specimens into one species is the general state of having five spots on the pronotum. In many populations there can be specimens without these markings although the majority of specimens tend to always have five black pronotal spots. Thus, with a single specimen it may not be possible to rely on this trait but with a series of specimens it is easy to determine the species as *P. liturata*, as it is now defined. There appears to also be some consistency in the characters of the ovipositor. In many of the Thailand specimens, the ovipositor appears remarkably like that figured by Lundblad (1933: fig. 42H) from Java (Indonesia) which is consistent with the ovipositor being a reliable character for species recognition (see Sublett and Cook 2015); however, a small number of specimens lack some or all of the secondary and tertiary teeth. Since the ovipositors of specimens from India, New Caledonia and Australia are unknown, there is still a question as to if specimens from these regions are actually *P. liturata.* Like *P. frontalis*, *P. liturata* could be a species complex. Both these species need additional study to determine their status.

*Ecology*: The habitat of *P. liturata* in Thailand is typical for the majority of pleids. This species was often found in ponds, in shallow water with vegetation.

### Paraplea melanodera

Taxon classificationAnimaliaHemipteraPleidae

Cook sp. nov.

31A8F396-448B-5857-BFDD-76558AA1C798

http://zoobank.org/B9B8C42C-2439-4A6D-9FC3-9F038C3CF863

**Figure 18.**
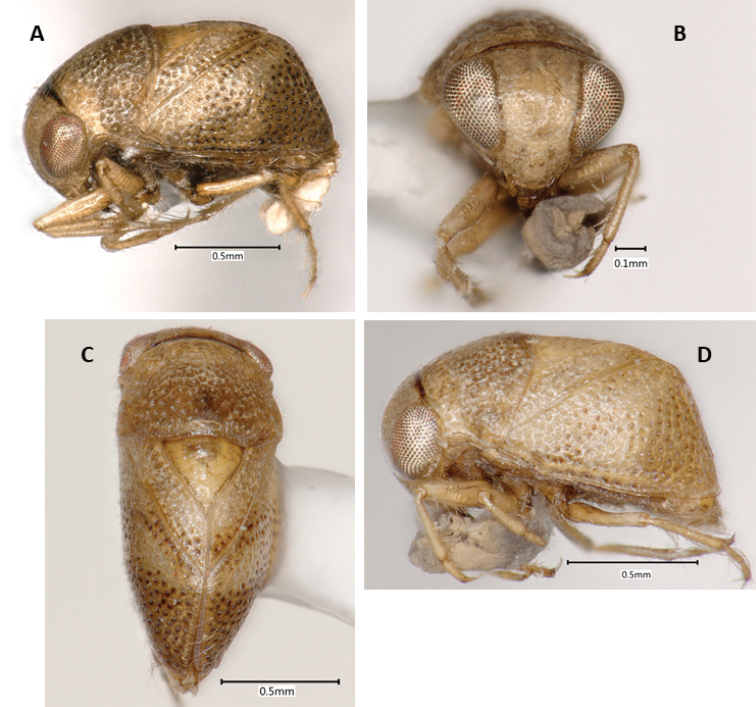
*Paraplea melanodera* sp. nov. **A** female in lateral view with typical coloration. **B** dorsal view of a light colored morph that shows dark banding and typical minimum amount of honeycombing. **C** frontal view of specimens in B. **D** female showing a lighter colored lateral view without dark hemelytral bands.

**Figure 19, 20.**
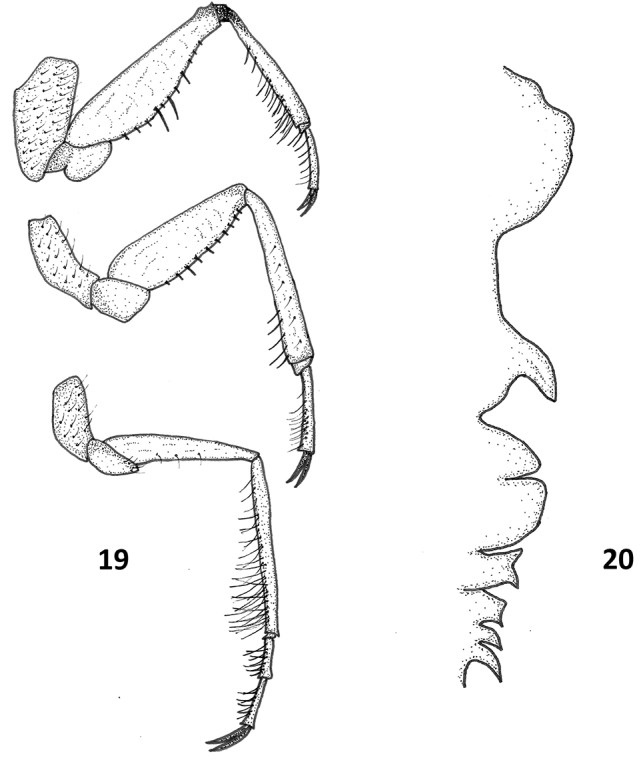
*Paraplea melanodera* sp. nov. **19** prothoracic leg at the top, mesothoracic leg in the middle and metathoracic leg below. **20** profile of the ventral keel with the anterior end (thoracic) to the top and ventral to the left.

**Figure 21.**
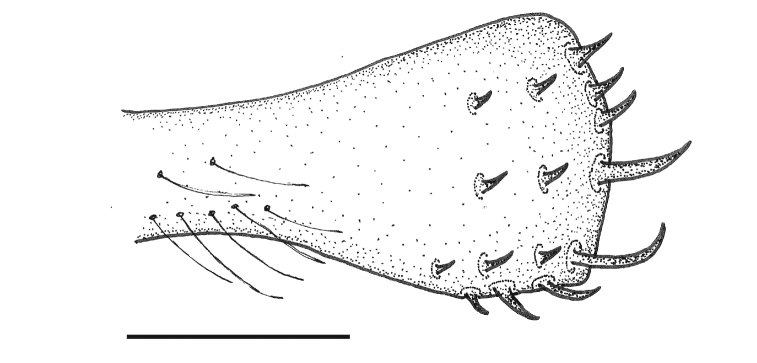
Ovipositor of *Paraplea melanodera* sp. nov. Scale bar: 0.05 mm.

**Figures 22, 23.**
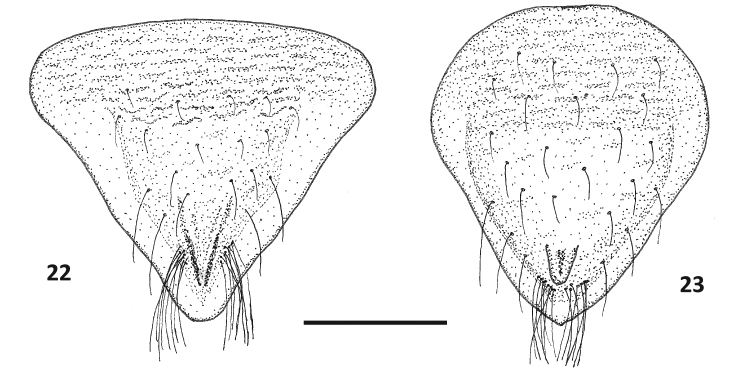
Genital plates of *Paraplea melanodera* sp. nov.: **22** female, **23** mele. Scale bar: 0.1 mm.

**Figure 24.**
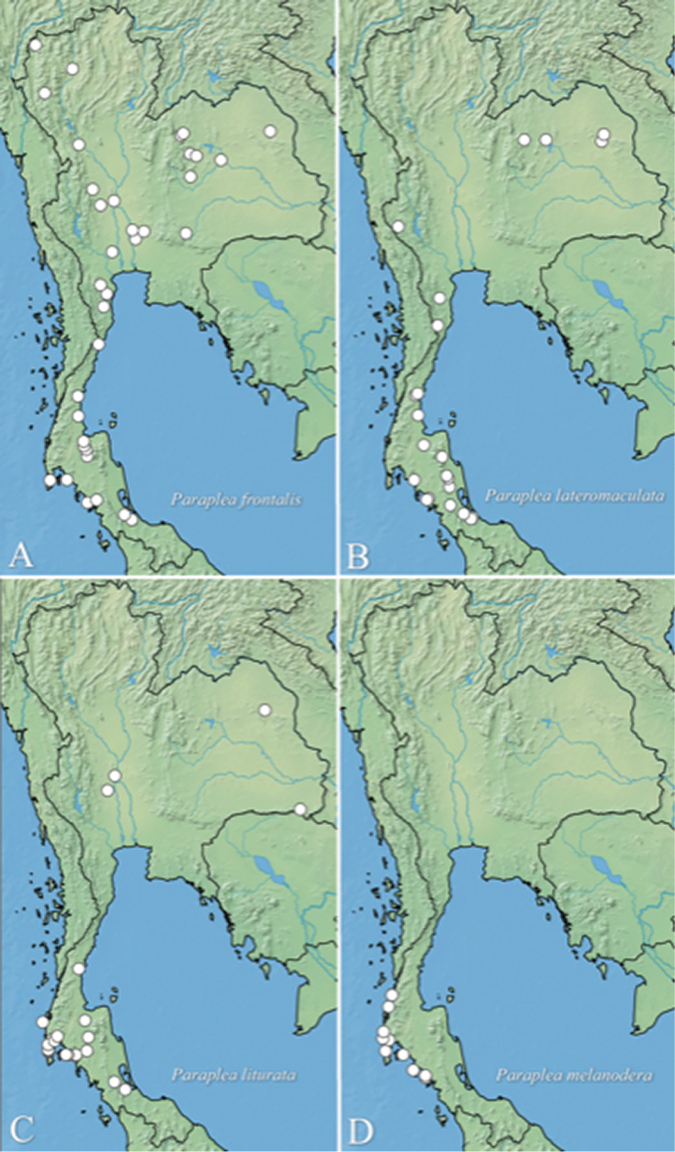
Distribution records in Thailand of **A***Paraplea frontalis***B***P. lateromaculata* sp. nov., **C***P. liturata***D***P. melanodera* sp. nov.

Figures 18 , 19, 20 , 21 , 22–23 , 24D

#### Description.

All measurements are in millimeters and were taken from 47 adult specimens from Thailand as reported in the distribution portion of this paper. Specimens used for this description are deposited at UMC and SHSU.

***Body size*.** Total length, 1.28–1.66 (average=1.49) (Fig. 18A, B, D).

***Color*.** Color may vary slightly among individuals (Fig. 18A, D) but all specimens with a dark brown to black band at back of vertex of head. Base color of body usually light tan to golden-tan with some darker brown markings (Fig. 18A); although, some specimens have a base color almost white (Fig. 18C, D). A few specimens with weak banding pattern of hemelytra (Fig. 18A, C); honeycombing present but often sparse and difficult to see in some specimens.

***Head*.** Head generally light brown, without markings except distinctive dark band posteriorly. Antenna three-segmented. Head width at widest point including eyes 0.62–074 (average 0.68), head width at narrowest point between eyes, 0.29–0.39 (average 0.34), OI 46–52 (average 49).

***Pronotum*.** Base color brown to light tan (Fig. 18C), usually with lighter colored honeycombing apparent although often not observable throughout; most specimens without dark spots; without distinct humeral or lateral bulges: punctures present, ~ 0.02, spacing between punctures ~ 0.02; if honeycombing present, punctures located between honeycomb bars (Fig. 18C); pronotum width 0.66–0.90 (average 0.78); pronotum length 0.42–0.57 (average 0.50); PI 56–73 (average 64).

***Wings*.** Hemelytra complete to posterior; punctures evenly dispersed with only small distance between punctures, punctures not in rows, ~ 0.02 diameter, evenly spaced (Fig. 18A, C, D); underlying honeycomb structure sometimes present; claval suture present (Fig. 18C, D); scutellum with distinct punctures, usually without darkened center, more widely spaced than other punctures, punctures not in apparent order; scutellum base color more yellow than hemelytra; hemelytra without spots near margin but sometimes with broad vertical bands (Fig. 18A, C); shape of hemelytra flat dorsally with posterior face at almost 45° angle (Fig. 18A, D); scutellum often slightly wider than long, sometimes as long as wide (Fig. 18C), length 0.28–0.39 (average 0.34); scutellum width 0.31–0.48 (average 0.39); SI 103–128 (average 115). Hind wings membranous, fully developed, completely concealed by hemelytra.

***Legs*.** Coxae and trochanters relatively very long compared to most pleid species (Fig. 19); hairs numerous on all coxae, relatively long and common on tibiae an tarsi, thickened on prothoracic tibia and tarsus; small spines on prothoracic and mesothoracic femora, small number longer on prothoracic femur; base of prothoracic tibia darker than other parts of leg. Typical leg measurements: prothoracic leg coxa 0.20, trochanter 0.11, femur 0.42, tibia 0.29, first tarsomere 0.03, second tarsomere 0.13, pretarsal claw 0.07; mesothoracic leg coxa 0.25, trochanter 0.08, femur 0.35, tibia 0.29, first tarsomere 0.02, second tarsomere 0.14, pretarsal claw 0.06; metathoracic leg coxa 0.19, trochanter 0.13, femur 0.39, tibia 0.42, first tarsomere 0.03, second tarsomere 0.14, third tarsomere 0.15, pretarsal claw 0.12; several long hairs along ventral side of trochanter, femur, tibia and tarsus, especially along hind tarsus where some hairs reach 0.18.

***Median ventral keel*.** Thoracic portions distinctively shaped but often hidden by enlarged coxa; prosternal keel broadly rounded, with irregular edges; mesosternal keel narrow, distinctive finger-like projection posteriorly; metathoracic keel segment somewhat rounded with prominent cleft towards center, sometimes appearing to almost overlap first abdominal section; abdominal keel I somewhat rectangular, with distinct tooth, abdominal keel II somewhat square, posterior tooth, abdominal keel III and IV shaped like posteriorly projecting teeth, IV longer than III (Fig. 20).

***Characters of female*.** Ovipositor expanded apically (Fig. 21); five distinct teeth along posterior border (apical row), two large teeth in ventral half, three smaller teeth in dorsal half; three teeth on ventral margin, decreasing in size basally. Two rows of three teeth each away from apex, three primary and three secondary; most specimens with one tertiary tooth; several hairs along basal region of gonapophysis 1 that can be seen under high magnification. Subgenital plate triangular, slightly wider than long (Fig. 22), width ~ 0.42, length ~ 0.37; central anterior area of plate raised above other parts, with v-shaped sub-apical prominence; rugose in basal one third; central region towards apex mildly rugose; two distinct tufts of longer hairs on each side of center near apex; other single hairs present, especially toward apex.

*Characters of male*: Aedeagus bulbous and somewhat asymmetrical in the typical fashion of family; operculum (subgenital plate), longer than wide (Fig. 23); width ~ 0.29, length ~ 0.33, rugulose in basal one third but only lightly rugose anterior to that region; central finger-like projection in center towards apex; central region raised above marginal areas; long hairs clumped near apex, other single hairs throughout.

#### Distribution.

*Paraplea melanodera* appears to be a species found only in peninsular Thailand along the west coast (Fig. 24D). Most of the known specimens are from ponds near the beach, thus it may be an endemic species to this region of Thailand.

#### Type material examined.

***Holotype***: female, Thailand: **Trang Province**, Amphur Sikao, pond at Chao Mai Beach, 7°26.842'N, 99°20.647'E, 3 m, 9 I 2006, Vitheepradit and Prommi, L-907 (UMC). ***Paratypes*** (10 total): Thailand: **Krabi Province**: Amphur Mueang, Khlong Muang Beach, 8°02.979'N, 98°45.540'E, 13 m, 8 I 2006, Vitheepradit and Prommi, L-901 (3 paratypes UMC, 2 paratypes SHSU). **Phang Nga Province**: Amphur Takua Pa, Nang Tong Beach, pond, 8°38.906'N, 98°14.833'E, 16 m, 4 I 2006, Vitheepradit, Sites and Prommi, L-883 (2 paratypes UMC). **Ranong Province**: Laem Son National Park, pond in front of officers house, 9°36.118'N, 98°28.074'E, 6 m, 2 VIII 2005, Vitheepradit, Prommi and Simpson, L-838 (1 paratype UMC); Laem Son National Park, pond near headquarters, 9°36.247'N, 98°28.005'E, 6 m, 3 I 2006, Vitheepradit, Sites and Prommi, L-875 (1 paratype UMC); Laem Son National Park, pond near headquarters, 9°36.247'N, 98°28.002'E, 6 m, 7 VI 2006, Vitheepradit, Sites and Prommi, L-922 (1 paratype UMC).

#### Additional material examined.

Thailand, **Krabi Province**: Amphur Ko Lanta, Khlong Dao Beach pond, 07°38.662'N, 99°01.395'E, 10 m, 9 VIII 2005, Sites, Vitheepradit, Simpson and Prommi, L-865 (5 specimens UMC); Amphur Mueang, Khlong Muang Beach, 8°02.979'N, 98°45.540'E, 13 m, 8 I 2006, Vitheepradit and Prommi, L-901 (12 specimens UMC, 1 specimen SHSU). **Phang Nga Province**: Amphur Khura Buri, Aow Kuey Beach, pond, 9°18.005'N, 98°22.798'E, 5 m, 7 VI 2006, Sites, Vitheepradit and Prommi, L-924 (2 specimens UMC); Amphur Takua Pa, Nang Tong Beach pond, 8°38.906'N, 98°14.833'E, 16 m, 4 I 2006, Vitheepradit, Sites and Prommi, L-883 (2 specimens UMC); Amphur Takua Pa, Tumbon Bang Nai Si, Ban Bang Yai, 8°25.950'N, 98°23.192'E, 20 m, 8 VI 2006, Vitheepradit, Sites and Prommi, L-927 (2 specimens UMC); Khao Lampi-Hat Thai Mueang National Park, pond near beach, 8°28.312'N, 98°13.672'E, 1 m, 2 VI 2005, Vitheepradit and Prommi, L-824 (1 specimen UMC). **Phuket Province**: Amphur Thalang, Jig peat swamp, 8°08.772'N, 98°17.892'E, 23 m, 10 VI 2006, Vitheepradit, Sites and Prommi, L-942 (1 specimen UMC). **Ranong Province**: Laem Son National Park, pond in front of officers house, 9°36.118'N, 98°28.074'E, 6 m, 7 VI 2006, Vitheepradit, Sites and Prommi, L-923 (1 specimen UMC); Laem Son National Park, pond near headquarters, 9°36.247'N, 98°28.005'E, 6 m, 2 VIII 2005, Vitheepradit, Prommi and Simpson, L-837 (2 specimens UMC); Laem Son National Park, pond near headquarters, 9°36.247'N, 98°28.005'E, 6 m, 7 VI 2006, Vitheepradit, Sites and Prommi, L-922 (5 specimens UMC). **Trang Province**: Tumbon Mai Fard, Ban Klong Maeng, pond, 7°30.170'N, 99°20.541'E, 6 m, 10 I 2006, Vitheepradit and Prommi, L-908 (4 specimens UMC).

#### Etymology.

The specific epithet combines two Greek roots, *melano*- meaning black and –*dero* meaning the neck. Thus, the name refers the distinct dark line at the back of the head, which is distinctive of this species and a character not found in other members of the genus.

#### Discussion.

The distinctive character of *P. melanodera* sp. nov. is the dark band at the posterior margin of the head. This character is seen in all specimens of this species and is not observed in any other species of *Paraplea*. *Paraplea melanodera* sp. nov. also has a raised central portion of the subgenital plate in both sexes and is most pronounced in the mele. Other species of *Paraplea* in Southeast Asia do not have this character state. The coxae of *P. melanodera* sp. nov. are quite long. *Paraplea lateromaculata* sp. nov. also has long coxae, but are comparably shorter than those of *P. melanodera* sp. nov. Spines on the metathoracic femur are not common in *Paraplea* but are present in both *P. melanodera* sp. nov. and *P. liturata*. *Paraplea melanodera* also has spines on the prothoracic femur, including a couple that are longer than the others. Characters of the ventral keel, ovipositor, and subgenital plates also have distinct differences compared to other species of *Paraplea*.

In a study to determine the recovery of the lentic insect community following the Indian Ocean tsunami of 2004, Sites & Vitheepradit (2010) sampled ponds along the Thai coastline at four time intervals, including beginning five months after the tsunami, which marked the end of the dry season. *Paraplea melanodera* sp. nov. was collected in 11 of the 12 ponds inundated by the tsunami, including during the first sampling period, and in only two of the ten un-inundated reference ponds. The mean conductivity of all inundated ponds over all dates from which *P. melanodera* sp. nov. was collected was 1,714 μS, including one at 13,040 μS. Conductivity of the reference ponds further inland was < 100 μS and Indian Ocean seawater was over 41,000 μS. Thus, the waterbodies in which *P. melanodera* sp. nov. occurred had distinctly elevated levels of salinity. It is likely that *P. melanodera* sp. nov. also occurs further north and south along the coastlines to Burma and Malaysia.

#### Distribution of *Paraplea* in mainland Southeast Asia.

*Paraplea frontalis* is one of the most widespread species of the genus and is prominent in Southeast Asia and beyond. In addition to the records reported here, *P. frontalis* has also been reported in other studies. Nieser (2004) stated that he had seen specimens of *P. frontalis* from Thailand, but these remain unpublished. The first published record of *P. frontalis* in Thailand did not appear until 2006 when it was reported from material collected in 1999 in Sakon Nakhon Province (Chen et al. 2006), which do not include those eluded to by Nieser (2004) (Nico Nieser pers. comm.). Lundblad (1933) described *P. quinquemaculata* (now a synonym of *P. frontalis*) from specimens from northern Sumatra. This region is directly west of mainland Malaysia and is considered maritime Southeast Asia, although with the short geographic distance across the Andaman Sea, it is not unexpected that these regions share species. Lundblad (1933) also reported two specimens of *P. frontalis* from Lake Toba, northern Sumatra and from East Java. Both of these specimens are in maritime Southeast Asia. Fernando (1961) collected *P. frontalis* (reported as *P. quinquemaculata*) at lights from Tanjong Karang, Selangor (Malaysia), which is close to the location of the Chapman specimens reported here. Fernando and Cheng (1974) mentioned that *P. frontalis* (reported as *P. quinquemaculata*) was collected once from a pond in Singapore but also stated that this species had never been collected in Malaya (Malaysia) even though Fernando (1961) had previously reported it from this region. Nieser and Chen (1999) reported on numerous collections of *P. frontalis* from Sulawesi and Sumatra (Indonesia). Esaki (1940) reported four specimens of *P. frontalis* from Chusan, which is an island off the southeastern coast of China. This region is not considered part of the political designation of Southeast Asia but is geographically close and this record would not be unusual with a mainland Southeast Asia species distribution. Esaki (1940) also stated that *P. frontalis* is widely distributed in India, Indochina, China, Java, Sumatra, Nicobar Islands and Formosa. However, no specimen data or source of that information was provided, nor did he mention any other Pleidae from this region. Esaki’s distribution could include data from several species, or it might include undocumented distribution records for *P. frontalis*. Distant (1910) used specimens from Calcutta and Madhupur, India to describe *Plea pelopea*, which is now a synonym of *P. frontalis*. Hafiz and Pradhan (1947) also reported *P. frontalis* from the nearby locality of Patnagarh, India and further north at Gait Sarovar, Bolangir, India. These areas of India share many species with a mainland Southeast Asia distribution; thus, these records might not be considered unusual. Benzie (1989) found *P. frontalis* from waterholes in Yala National Park in southeastern Sri Lanka.

It is uncertain what factors influence the distribution of *P. frontalis*. The limited number of collections that represent our knowledge of this species suggest that it is most common in mainland Southeast Asia, but its distribution extends to the north into eastern Asia in China and Taiwan, west to India and Sri Lanka, and south to maritime Southeast Asia on islands of Indonesia. This paper represents the only data where an area was more thoroughly collected, although even this effort provides fewer than 500 specimens. When these collections are plotted on a distribution map (Fig. 24A), there is no perceptible indication of reasons for this distribution. In fact, with more collections, *P. frontalis* may be found throughout nearly all provinces of Thailand.

Another factor to consider is that what is reported as *P. frontalis* could be a species complex. This would not pertain to the distribution shown in Fig. 24A but could be a factor in reports in other areas of its distribution. There are noted morphological differences among specimens of *P. frontalis* from different parts of its range, including size differences, some differences in markings and other minor morphological variation. More study and many more specimens are needed to sort out this situation, probably including modern molecular comparisons of specimens from these various regions. Until this future study, *P. frontalis* will be considered a variable species with a wide distribution.

*Paraplea liturata* is widely distributed in Thailand and some predict that it could have the largest distribution of any species in the genus (Lundblad 1933, Fernando 1961). Its distribution in Thailand (Fig. 24C) does not appear as extensive of that of *P. frontalis* but it is a common inhabitant within a wide geographic distribution. Fernando and Cheng (1974) collected *P. liturata* at several locations in Malaysia and recorded it as being “fairly common” in this region. Nieser and Chen (1999) likewise found it in several locations in Indonesia as well as documenting the species in the Philippines.

*Paraplea liturata* does not appear to be restricted to any general habitat type or biogeographical region. Chen et al. (2006) stated that it was “common throughout Thailand in vegetation rich stagnant waters.” The present study also indicates that the species occurs in ponds and slow-moving streams, both containing aquatic vegetation. Anderson and Weir (2004) reported *P. liturata* from Western Australia and the Australian Northern Territory, areas biogeographically quite different from Thailand and Malaysia, but did not comment on the specifics of the collection sites.

The distribution of *P. lateromaculata* sp. nov. (Fig. 24B) is very similar to that of *P. liturata* (Fig. 24C) in Thailand, including collection sites in common between these species. Both of these species were common in the peninsular region of Thailand.

The documented distribution of *P. melanodera* sp. nov. includes only the central part of peninsular Thailand (Fig. 24D). Even more restrictive is that it was collected only from small ponds near beaches along the coastline. The apparent halophilic nature of *P. melanodera* sp. nov. is well-documented because all specimens were collecting during the tsunami study of Sites & Vitheepradit (2010). The other three species of *Paraplea* also were collected during the study; however, these were mostly from un-inundated reference ponds. More specifically, *P. lateromaculata* sp. nov. was collected in two reference ponds, including during all four sampling periods. *Paraplea liturata* was collected in all reference ponds over all sampling periods as well as a single individual from an inundated pond on the first sampling date, which we consider an adventitious occurrence. *Paraplea frontalis* was collected in two inundated ponds with elevated levels of salinity on the last sampling date, which was 17 months after the tsunami. Because eight and 28 specimens were collected in those two ponds, its occurrence was not adventitious; thus, *P. frontalis* appears to have a tolerance for salinity, but is not as adept at dispersing as is *P. melanodera* sp. nov.

## Supplementary Material

XML Treatment for Paraplea frontalis

XML Treatment for Paraplea lateromaculata

XML Treatment for Paraplea liturata

XML Treatment for Paraplea melanodera
